# Simultaneous adsorption of ammonia nitrogen and phosphate on electro-assisted magnesium/aluminum-loaded sludge-based biochar and its utilization as a plant fertilizer

**DOI:** 10.1371/journal.pone.0311430

**Published:** 2024-10-25

**Authors:** Qi Wang, Chu-Ya Wang, Heng-Deng Zhou, Dong-Xin Xue, Xiao-Lu Xiong, Guangcan Zhu

**Affiliations:** School of Energy and Environment, Southeast University, Nanjing, China; Southwest University, CHINA

## Abstract

Herein, Mg/Al-loaded sludge-based biochar was prepared via electro-assisted impregnation. The structure and chemical analysis of modified sludge-based biochar (MgSBC-0.5(@Al) showed that the material was loaded with MgO and Al_2_O_3_. The specific surface area of MgSBC-0.5(@Al) was 11.27 times higher than that of unmodified sludge-based biochar (SBC). The simultaneous adsorption performance of MgSBC-0.5(@Al for ammonia nitrogen (NH_4_^+^–N) and phosphate phosphorus (PO_4_^3−^–P) was studied. The maximum adsorption capacities of MgSBC-0.5(@Al for NH_4_^+^–N and PO_4_^3−^–P at 298 K were 65.19 and 92.10 mg·g^−1^, respectively, 4.45 and 6.28 times higher than those of SBC. The external and internal elemental compositions of the modified and unmodified biochar specimens were quantitatively characterized using inductively coupled plasma mass spectrometry, X-ray photoelectron spectroscopy, and X-ray fluorescence spectrometry. The results emphasized the importance of Mg-loading for NH_4_^+^–N and PO_4_^3−^–P capture. MgO was mainly loaded on the surface of biochar, enabling adsorption through chemical reactions. Analysis showed that the adsorption of NH_4_^+^–N and PO_4_^3−^–P on the modified biochar proceeded simultaneously through multiple mechanisms. Particularly, the adsorption of NH_4_^+^–N and PO_4_^3−^–P occurred through the precipitation of struvite and physical adsorption, with PO_4_^3−^–P also adsorbed through the formation of MgHPO_4_ and CaHPO_4_. Other data indicated that Al, Ca, and Fe had a trapping effect on the adsorbate. Importantly, the biochar after adsorption could be used as a soil amendment.

## Introduction

With the recent surge in industrialization, the size of sewage treatment facilities has rapidly increased. The production of sludge, as a by-product of sewage treatment plants, has also been gradually increasing. In Europe, the annual production of sludge can reach 10 million tons (in dry weight) [1]. The degradation rate of organic matter in sludge after anaerobic fermentation is only 40%–60%, with a considerable amount of organic matter remaining in the fermentation residue [2]. The discharge of sewage sludge containing nitrogen and phosphorus detrimentally affects water quality, leading to the eutrophication of water bodies [3]. Therefore, there is a pressing need to develop efficient and cost-effective methods for treating wastewater and sludge.

Biochar (BC) is a porous, loose, aromatic solid product formed through the pyrolysis of biomass under limited oxygen conditions. BC has a certain amount of surface functional groups, large specific surface area, wide availability of required raw materials, and low cost. Additionally, BC can adsorb pollutants from water [4]. Agricultural waste (such as straw, sawdust, sugarcane bagasse, and rice bran) and animal manure can be used as raw materials for the production of BC [5]. Some studies have shown that BC prepared from sludge exhibits strong adsorption capacity for organic and metal pollutants. Pyrolysis substantially reduces the ecological toxicity of heavy metals in sludge BC, reducing the environmental risks of its application [6]. Compared to BCs based on other types of biomass, sludge BC can be used to cost-effectively adsorb N and P. Therefore, sludge BC has unique advantages over other BC materials in practical applications [7]. However, the functionality and adsorption capacity of BC prepared via conventional pyrolysis are limited, making it unsuitable for pollutant adsorption. Consequently, BC is chemically modified, including impregnation modification with single metals (Fe, Mg, etc.) [8–10] and pairs of metals (Mg/Al, Ca/Mg, etc.) [11,12]. Among the modified BCs, magnesium salt–modified biochar has high surface activity, anion fixation ability, and ion exchange ability. Magnesium-modified BC (Mg–BC) has been previously studied as a potential adsorbent for N and P. Mg–BC is prepared mainly through impregnation carbonization and carbonization impregnation. The modification of BC with Mg has been performed by soaking it in MgCl_2_ solution. However, the immersion time is long, usually more than 2 h [13]. Therefore, some researchers have proposed electro-assisted modification. Mg/Al bilayer metal–modified BC was prepared via an electro-assisted method using MgCl_2_ solution electrolyte and Al electrode, with an impregnation time of only 5 min. The formed compounds (MgO, spinel MgAl_2_O_4_, AlOOH, and Al_2_O_3_) evenly covered the surface of BC, exhibiting a highly organized and well-defined structure, resulting in improved PO_4_^3−^–P adsorption capability of BC.

However, such a modification produces toxic Cl_2_ gas and was employed only in the studies on the adsorption of PO_4_^3−^–P from sewage. However, in sewage, NH_4_^+^–N may co-exist with PO_4_^3−^–P. Moreover, the BC material used in the above study was prepared from seaweed, which is more expensive than sludge. Importantly, NH_4_^+^–N may accumulate in the sewage treatment plant owing to anaerobic sludge digestion. However, PO_4_^3−^–P and NH_4_^+^–N coexisting in solution may be removed using Mg^2+^. Therefore, sludge-based BC (SBC) has unique advantages over other BC materials in practical applications.

Therefore, the present study aimed to prepare and characterize a bimetallic-modified SBC, with modification performed using Mg salt solution as an electrolyte in an electro-assisted system. Additionally, simultaneous adsorption capabilities of the prepared material for NH_4_^+^–N and PO_4_^3−^–P in wastewater were determined. The results show that sludge can be modified within 10 min and pyrolyzed to obtain functionalized sludge-based BC (Mg/Al assembled composite) with a high surface area. The obtained results provide a valuable reference for the preparation of modified SBCs for the adsorption of NH_4_^+^–N and PO_4_^3−^–P. Additionally, the applicability of saturated adsorbents as soil amendments was also explored. Overall, the use of modified SBC is a green and novel method for recovering NH_4_^+^–N and PO_4_^3−^–P from sewage sludge.

## Materials and methods

### Preparation of materials

After anaerobic fermentation, the sludge was dried in an oven at 105°C to remove moisture content, ground to a fine powder (smaller than 200 mesh), and stored in a dry place.

Mg/Al-loaded SBC (MgSBC-0.5(@Al)) was prepared by immersing 2 g of dry sludge powder (named SP) in 0.5 mol·L^−1^ magnesium acetate solution (100 mL). Subsequently, the pH of the solution was adjusted to 3.0 using 0.25 M HCl and NaOH. Electro-assisted modification was performed using a power supply to apply the appropriate current density (fixed at 93.96 mA cm^−2^; programmable DC power supply, ODA, Korea). The anode and cathode rods of the electrochemical modification experimental device were both Al electrode, and a saturated calomel reference electrode was used. Aluminum electrodes had an effective surface area of 0.07065 cm^2^, and the separation between the electrodes was adjusted to 3 cm. The electro-assisted modification was conducted under constant stirring at 120 rpm for 10 min. The modified sludge was dried at 65°C, pyrolyzed at 500°C (heating rate: 5°C·min^−1^) for 2 h in a nitrogen (N_2_) environment, ground to a fineness of 200 mesh, and encapsulated in a container.

MgSBC-0.1(@Al), MgSBC-0.25(@Al), and MgSBC-1(@Al) were prepared following the same procedure as MgSBC-0.5(@Al but using different magnesium acetate concentrations during electro-assisted modification-0.1, 0.25, and 1 mol/L, respectively. MgSBC-0.5(@Al) was prepared by soaking SP in 0.5 mol·L^−1^ magnesium acetate and stirring at 120 rpm for 6 h. The drying, pyrolysis, and subsequent procedures were the same as in the preparation of MgSBC-0.5(@Al). The unmodified SBC specimen (named SBC) was prepared via thermal decomposition at 500°C (heating rate: 5°C·min^−1^) in N_2_ environment for 2 h.

All reagents were obtained from Aladdin Industrial Company (Shanghai, China). Solutions containing NH_4_^+^–N (measured in N units) and PO_4_^3−^–P at concentrations of 1000 mg·L^−1^ were prepared by dissolving appropriate amounts of NH_4_Cl and KH_2_PO_4_ in deionized water and diluting them to 1000 mL in a volumetric flask. Subsequently, the prepared stock solution was diluted to the desired concentration.

### Batch adsorption experiments

The effects of initial pH, concentration, contact time, and presence of other ions on the adsorption properties of the prepared materials were determined using the isotherm adsorption method. Adsorption solution containing 50 mg·L^−1^ of PO_4_^3−^–P and 100 mg·L^−1^ of NH_4_^+^–N was used in this and subsequent experiments. Modified BC (0.02 g) was added to 30 mL of the adsorption solution, the pH was adjusted to 7, and the mixture was shaken at a speed of 150 rpm. Then, a syringe filter with a pore diameter of 0.45 μM was used to separate the mixture. The quantities of NH_4_^+^–N and PO_4_^3−^–P adsorbed were calculated using Eq (1):

$$Q_{t}=\frac{V}{m}(C_{0}‐C_{t})$$ (1)

where *Q*_t_ (mg·g^−1^) is the amount of NH_4_^+^–N and PO_4_^3−^–P adsorbed by each gram of an adsorbent at time *t*, *C*_0_ (mg·L^−1^) denotes the initial concentrations of NH_4_^+^–N and PO_4_^3−^–P, *C*_t_ (mg·L^−1^) indicates the concentrations of NH_4_^+^–N and PO_4_^3−^–P at time *t*, (*C*_0_ − *C*_t_) is the change in the concentrations of NH_4_^+^–N and PO_4_^3−^–P, *m* (mg) is the mass of the adsorbent, and *V* (mL) is the solution volume. The concentrations of NH_4_^+^–N and PO_4_^3−^–P remaining in the solution can be calculated once the reaction reaches Eq (2).

$$Q_{e}=\frac{V}{m}(C_{0}‐C_{e})$$ (2)

where *Q*_e_ (mg·g^−1^) is the amount of NH_4_^+^–N and PO_4_^3−^–P adsorbed by each gram of the adsorbent, and *C*_e_ (mg·L^−1^) represents the concentrations of NH_4_^+^–N and PO_4_^3−^–P at equilibrium.

To construct adsorption isotherms, adsorption experiments were conducted using mixed adsorption solutions of different concentrations (0, 5, 10, 15, 25, 50, 100, 200, 250 and 400 mg·L^−1^). Adsorption experiments were conducted at 25°C, 35°C, and 45°C, and the pH of the solution was adjusted to 7 using HCl and NaOH. The formula is as stated:

$$Q_{e}=Q_{\max}K_{L}C_{e}/(1+K_{L}C_{e})$$ (3)

$$Q_{e}=K_{f}C_{e}^{1/n}$$ (4)

where *Q*_max_ (mg·g^−1^) represents the maximum adsorption capacity of the adsorbent, and *K*_f_ [mg·(g·(L·mg^−1^)1/n)^−1^] and *K*_L_ (L·mg^−1^) are the constants in the Freundlich and Langmuir models, respectively.

Adsorption kinetics were also investigated. Specifically, 50 mg·L^−1^ PO_4_^3−^–P and 100 mg·L^−1^ NH_4_^+^–N solutions were mixed and stirred at 35°C and 150 rpm. The pH of the solution was adjusted to 7. The concentrations of NH_4_^+^–N and PO_4_^3−^–P were measured by collecting samples at the designated time points. The adsorption of NH_4_^+^–N and PO_4_^3−^–P by the prepared materials was analyzed using the pseudo-first-order, pseudo-second-order, and Weber–Morris models. The corresponding equations are provided below.

$$\ln(Q_{e}‐Q_{t})=\ln Q_{e}‐k_{1}t$$ (5)

$$t/Q_{e}=1/(k_{2}Q_{e}^{2})+t/Q_{e}$$ (6)

where *k*_1_ (min^−1^) is the adsorption rate constant, *k*_2_ (g·mg^−1^·min^−1^) is the adsorption rate constant in the pseudo-second-order model.

$$Q_{t}=k_{\mathrm{id}}t^{1/2}+C$$ (7)

where *k*_id_ is the intra-particle diffusion rate constant (mg/(g·min^1/2^)), *t* (min) is the adsorption time, and *C* is a constant that depends on the width of the adsorption boundary layer.

The effect of pH on adsorption was determined at 35°C by changing the pH of the initial solution in the range of 3–11 using either HCl or NaOH. The solid–liquid ratio was maintained at 0.67 g·L^−1^.

Thermodynamic experiments were conducted at 25°C, 35°C, and 45°C to elucidate the natural occurrence and adsorption of NH_4_^+^–N and PO_4_^3−^–P. The Gibbs free energy (Δ*G*_0_; kJ·mol^−1^) was calculated based on the experimental data obtained at various temperatures, employing the equation that incorporates changes in enthalpy (Δ*H*_0_; kJ·mol^−1^) and entropy (Δ*S*_0_; kJ·mol^−1^·K^−1^).


$$\Delta G_{0}=\Delta H_{0}‐T\Delta S_{0}$$ (8)

$$\Delta G_{0}=‐RT\ln K_{C}$$ (9)

$$K_{C}=M_{W}\times 55.5\times 1000\times K_{L}$$ (10)

Here, *R* (8.314 J·mol^−1^·K^−1^) is the gas constant, *K*_C_ is the adsorption constant, and *T* (K) is the absolute temperature. The Langmuir constant, denoted as *K*_L_, is the equilibrium constant for adsorption, while *M*_W_ (mol) is the quantity of the adsorbent. Using Eqs (8–10), the intercept and slope of the temperature curve of Δ*G*_0_ were determined as Δ*S*_0_, Δ*H*_0,_ and *T*.

To assess the relative competitiveness of various ions, the adsorption solution was supplemented with anions (Cl^−^, HCO_3_^−^, SO_4_^2−^, and CO_3_^2−^) and cations (Na^+^, Fe^3+^, and Ca^2+^) that are commonly found in wastewater. Their concentrations were varied in increments of 50 mmol·L^−1^ in the range of 0–250 mmol·L^−1^.


$$D=\frac{Q_{e}‐Q_{a}}{Q_{e}}\times 100\%$$ (11)

### Pot experiments

The potential of MgSBC-0.5(@Al) as a fertilizer was investigated using a pot experiment. The soil was collected from 0–20 cm topsoil at Southeast University (China). The basic properties of soil are shown in Table 1.

**Table 1:** Basic properties of soil.

	Moisture content	pH	CEC	Organic matter	Total phosphorus	Total nitrogen	Available Phosphorus	Hydrolyzable nitrogen
Primitive soil	%	/	(cmol·kg^-1^)	(g·kg^-1^)	(g·kg^-1^)	(g·kg^-1^)	(mg·kg^-1^)	(mg·kg^-1^)
	11.7	7.72	18.86	18.9	0.56	0.88	24.20	49

MgSBC-0.5(@Al) saturated with NH_4_^+^–N and PO_4_^3−^–P (hereafter referred to as MgSBC-0.5(@Al)–NP) was added to the soil at 0.5% of soil dry weight. The leaching process was conducted using the methodology specified by the United States Environmental Protection Agency (USEPA 2014). Mung beans were used as test plants in this experiment. MgSBC-0.5(@Al)–NP, SBC–NP, commercial nitrogen phosphorus fertilizer and SP (0.5 g) were thoroughly mixed into 100 g soil and added to the pot (group A, B, D and E), whereas group C (control) was grown on soil without any additions. Five mung beans were planted in each pot and allowed to grow in a greenhouse at a temperature of around 25°C. Each grid was watered with 2.5 mL of tap water every day. The growth of mung bean sprouts was recorded. On the 14th day, the plants were selected for physical properties determination. The germination rate (GP; %) of mung bean sprouts was calculated using Eq (12).

$$\mathrm{Germination}\;\mathrm{percentage}(\mathrm{GP})=\frac{n}{N}\times 100\%$$ (12)

where *n* is the number of seeds germinated on the fourteenth day, and *N* is the total number of seeds.

### Characterization and analytical method

Mo–SS anti-spectrophotometry was employed, in addition to a spectrophotometer (752 N-spectrophotometer; China). The pH of the solution was determined using a pH meter (pHS-3C, China). The N_2_ adsorption–desorption isotherms were determined using the Quantachrome AUTOSORB IQ (United States). The specific surface area (*S*_BET_) and pore characteristics were measured using the Barrett–Joyner–Halenda method. The morphology and microstructure of the prepared materials were analyzed using scanning electron microscopy (SEM; Gemini 300, Zeiss, Germany). Surface elemental analysis was performed using energy-dispersive X-ray spectroscopy (EDS) at the same locations where SEM was performed. The microstructure and crystal structure were analyzed through X-ray diffraction (XRD; D2 phaser, Bruker, Germany). The composition was analyzed within the 2θ range of 10°–80° captured at the position. The Nickel IS10 catalyst was analyzed through Fourier transform infrared spectroscopy (FTIR) in the wavenumber range of 4000–400 cm^−1^. X-ray photoelectron spectroscopy (XPS; K-Alpha, Thermo Scientific, USA) was performed using an Al K α source (hv = 1486.6 eV). Additionally, the pressure within the sample chamber should not exceed 2.0× Perform XPS detection at a pressure of 10^−7^ mbar. The elemental composition of the bulk phase of the samples was determined using inductively coupled plasma (ICP)-optical emission spectrometry (OES)/mass spectrometry (MS; Fisher iCAP PRO) and X-ray fluorescence (XRF; SHIMADZU XRF-1800). SPSS statistics 27 was used for significance analysis. Nitrogen and phosphorus contents in mung bean sprouts in five groups (14 days) were determined by Kjeldahl nitrogen analyzer and inductively coupled plasma (ICP)-optical emission spectrometry (OES)/mass spectrometry (MS; Fisher iCAP PRO).

## Results and discussion

### Characterization and morphology

**Fig 1:**
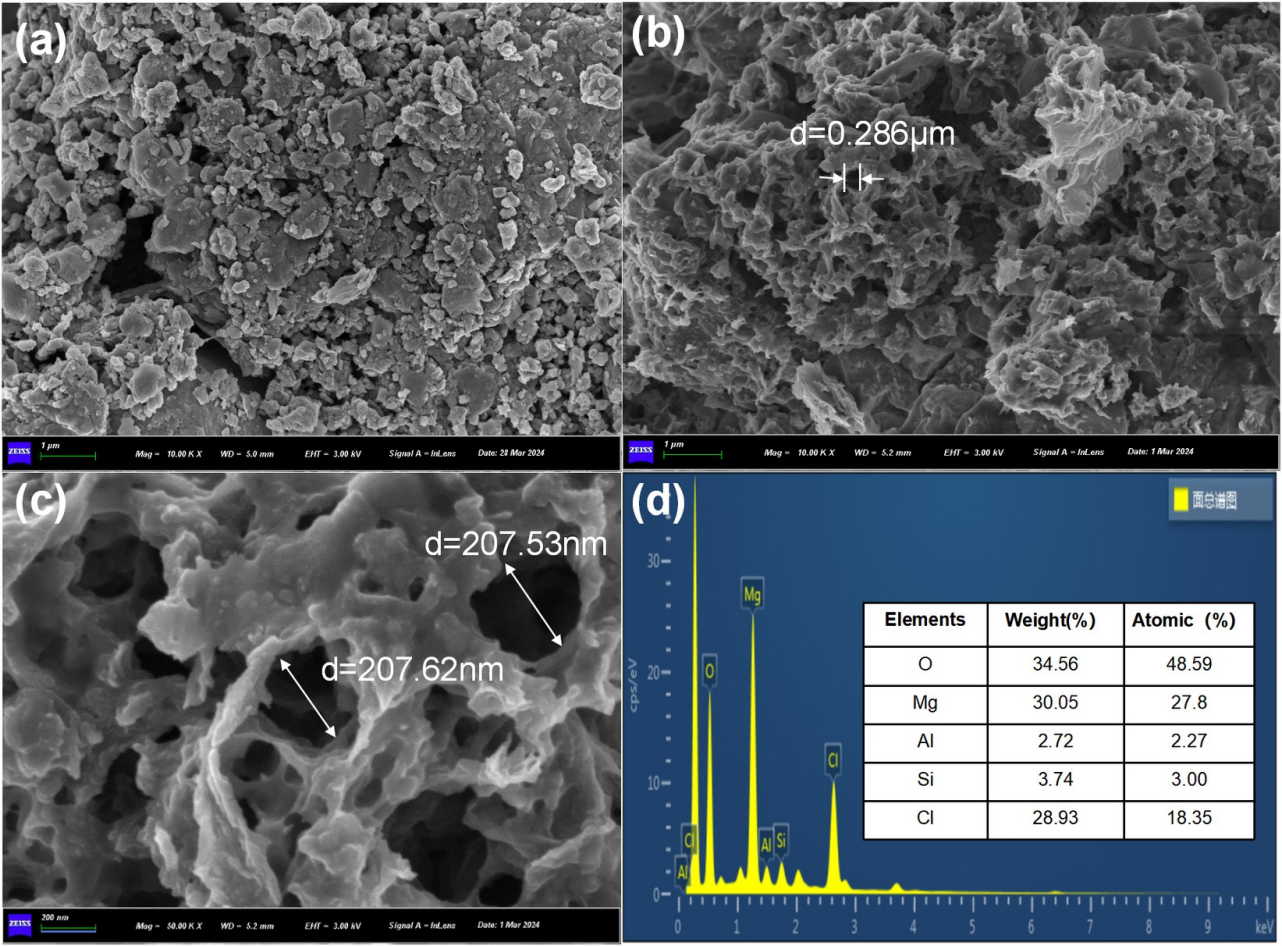
(a) SEM images of SBC, SEM images of MgSBC-0.5(@Al) at different expansion ratios (b-c) and (d) EDS spectra of MgSBC-0.5(@Al) corresponding to SEM images.

Fig 1 shows the SEM images of the prepared BC specimens, with MgSBC-0.5(@Al) exhibiting a rougher porous structure than SBC (Fig 1A). Additionally, Fig 1B and 1C show that the pore size of MgSBC-0.5(@Al) is greater than 200 nm, providing additional adsorption sites. Fig 1D shows the energy spectrum of MgSBC-0.5(@Al), demonstrating that the sample mainly consists of Mg, Al, Si, Cl, and O [14].

**Fig 2:**
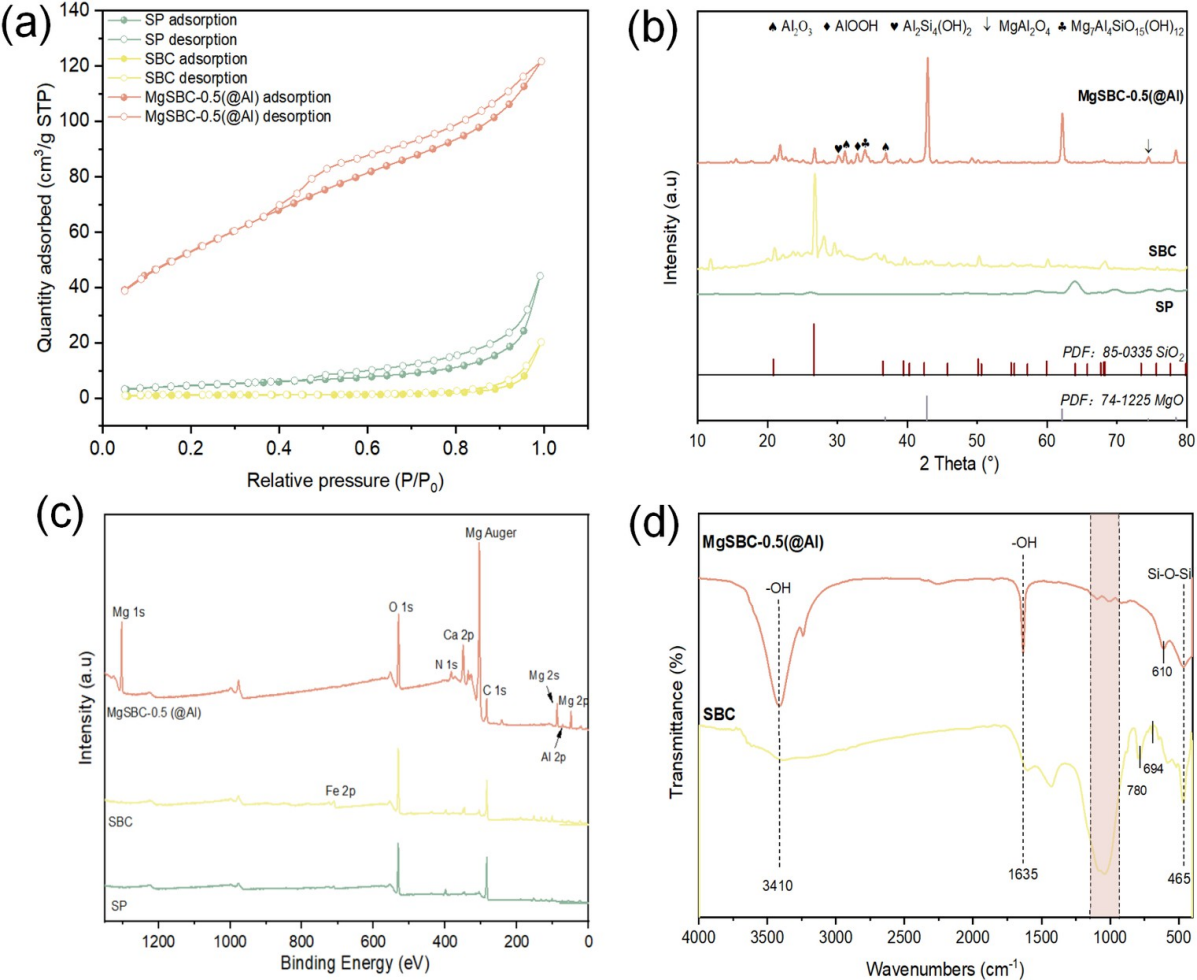
(a) Adsorption and desorption curve, (b-d) XRD patterns, XPS spectra and FTIR spectra of SP, SBC and MgSBC-0.5(@Al).

N_2_ adsorption–desorption isotherms were obtained for SP, SBC, MgSBC-0.5(@Al), and MgSBC-0.5 to determine their *S*_BET_, total pore volume, and average pore diameter (APD; Table 2). The *S*_BET_ of the four materials is 4.59, 16.76, 188.86, and 10.44 cm^2^·g^−1^, respectively. The *S*_BET_ and total pore size of MgSBC-0.5(@Al) are 11.27 and 5.94 times higher than those of SBC, respectively. The APDs of the four materials are within 2–50 nm, making them mesoporous materials [15]. In particular, the *S*_BET_ of MgSBC-0.5 is substantially smaller than that of MgSBC-0.5(@Al), indicating that the electro-assisted modification increased the *S*_BET_ of BC. However, the APD of MgSBC-0.5(@Al) is low, even though the pore size on the surface of the material is more than 200 nm. Such results were attributed to the blocking of a portion of the pores by the metal oxide or the collapse of the pore structure during high-temperature calcination. The removal of certain substances from the sludge would open some blocked internal pore structures, leading to a reduction of APD. Subsequently, N_2_ adsorption–desorption isotherms were obtained at 77 K. Based on the IUPAC classification, the N_2_ adsorption curves of SP, SBC, and MgSBC-0.5(@Al) are all type IV adsorption isotherms, with an H_3_ hysteresis loop (Fig 2A). At the same time, MgSBC-0.5(@Al) shows no substantial limit adsorption in the *P*/*P*_0_ range of 0.9–1.0. The above indicates that the material contains slit-like pores and has a flake-particle-aggregation pore structure [16,17]. Research found that electro-assisted modification substantially increased the pore size and *S*_BET_ of BC as well as enriched its pore structure. These findings have important implications for the practical application of BC in adsorption.

**Table 2:** Comparison of S_BET_, Total pore volume, and APD for SP, SBC, MgSBC-0.5 and MgSBC-0.5(@Al).

Parameters	Adsorbent			
	SP	SBC	MgSBC-0.5(@Al)	MgSBC-0.5
S_BET_ (m^2^·g^−1^)	4.59	16.76	188.86	10.44
Total pore volume (cm^3^·g^−1^)	0.031	0.037	0.19	0.044
APD (nm)	27.37	8.86	3.95	16.98

The XRD results are shown in Fig 2B, indicating that SP and SBC are mainly composed of SiO_2_. For MgSBC-0.5(@Al), the diffraction peaks observed at 36.86° (111), 42.83° (200), 62.30° (220), 74.69° (311), and 78.63° (222) were ascribed to MgO. Spinel MgAl_2_O_4_ (PDF # 21–11052), AlOOH (PDF # 21–1307), Al_2_O_3_ (PDF # 10–0425), and Mg_7_Al_4_SiO_15_(OH)_12_ (PDF # 47–1866) were also detected. The presence of diffraction peaks that were difficult to identify indicates that the specimens contained small amounts of other composite minerals, such as Mg/Al–Si composite minerals [18,19].

The elemental composition was investigated using XPS. According to the XPS results in Table 3 and Fig 2C, the surface of MgSBC-0.5(@Al) is mainly composed of C, O, N, Si, Al, Ca, and Fe. The contents of Mg and Al in MgSBC-0.5(@Al) are 28.08% and 6.24%, indicating that the oxides of Mg and Al were loaded on the SBC [19]. The loading of Al_2_O_3_ was beneficial for the formation of porous structures in biochar. The presence of Al^3+^ in solution could precipitate with PO_4_^3—^P through strengthened chemical bonds. This might improve the adsorption capacity of MgSBC-0.5(@Al) for PO_4_^3—^P [20]. SP has been reported to contain a small amount of Ca and Fe, which could form amorphous CaCO_3_ and FePO_4_) with PO_4_^3−^–P, benefiting the adsorption of PO_4_^3−^–P [21]. The ICP–MS analysis of the entire sample of MgSBC-0.5(@Al) shows (Table 3) that Mg accounts for 30.3% of the sample, slightly higher than the 28.08% obtained through XPS, indicating that Mg is mainly loaded on the surface of the sample (rather than in the bulk). This might reflect the adsorption of NH_4_^+^–N and PO_4_^3−^–P, during which Mg dissolves from the surface of the adsorbent into the solution during exposure or rearranges on the surface after initial dissolution, forming local mineral complexes (MgHPO_4_). The formation of these complexes can lead to the exposure of carbon lattice, as shown in SEM images (Fig 1B) [22].

**Table 3:** The elemental composition (atomic%) measured by XPS and ICP-MS.

Analysis methods	Sample	C	O	Si	Mg	Al	Ca	Fe	P	N	Cl
XPS	SP	61.05	30.24	4.44	0.52	/	1.34	1.06	1.03	0.33	/
	SBC	47.70	36.14	7.44	0.91	/	2.97	2.18	1.83	0.83	/
	MgSBC-0.5(@Al)	14.34	23.69	0.43	28.08	6.14	4.56	9.23	2.01	0.49	11.03
ICP-MS	MgSBC-0.5(@Al)	/	/	/	30.3	6.3	/	/	/	/	

Fig 2D shows the FTIR spectra of SP, SBC, and MgSBC-0.5(@Al) in the wavenumber range of 4000–400 cm^−1^. The peak observed at a wavelength of 465 cm^-1^ in SBC was attributed to the Si-O-Si or Al-O-Al vibration. Symmetric and antisymmetric tensile vibrations of Si–O–Si chains are represented by peaks at 694 and 780 cm^−1^, respectively [23,24]. The peaks at 3410 and 1635 cm^−1^ in MgSBC-0.5(@Al) correspond to the stretching vibration of interlayer hydrogen bonding groups and water molecules [25,26]. This indicated that MgSBC-0.5(@Al) may contain bound water in its structure. The peak at 610 cm^−1^ observed in the visible band corresponds to the metal–oxygen bonds (M–O and M–O–M, where M represents either Mg or Al) [27]. Additionally, MgSBC-0.5(@Al) exhibits substantially different absorption peaks than SBC. In region I, a wider peak is observed for SBC, whereas MgSBC-0.5(@Al) shows a number of narrower peaks. This change may have occurred because of the transformation of Si–O–Si bonds into Si–O–Mg and Si–O–Al bonds, which have greater bond lengths and smaller bond angles than the Si–O–Si bonds [28].

### Adsorption isotherm and kinetics

Magnesium acetate solutions with concentrations of 0.1, 0.25, 0.5, and 1 mol·L^−1^ were prepared. The adsorption capabilities of MgSBC-0.1(@Al), MgSBC-0.25(@Al), MgSBC-0.5(@Al), and MgSBC-1(@Al)) for NH_4_^+^–N and PO_4_^3−^–P were compared using S1A Fig MgSBC-0.5(@Al) shows the highest adsorption capacities for NH_4_^+^–N and PO_4_^3−^–P at 308 K, 28.22 and 58.73 mg·g^−1^, respectively. The four materials were characterized by XRD. It can be seen from S1B Fig that the peak of MgO in MgSBC-0.5(@Al) is higher than that of the other three materials, indicating that MgSBC-0.5(@Al) is loaded with more MgO. Mg^2+^ might play a role in the formation of struvite crystals. This transformation was not beneficial for the adsorption of NH_4_^+^–N and had minimal impact on the adsorption of PO_4_^3−^–P. Therefore, setting a limit for Mg-loading was essential [29].

**Fig 3:**
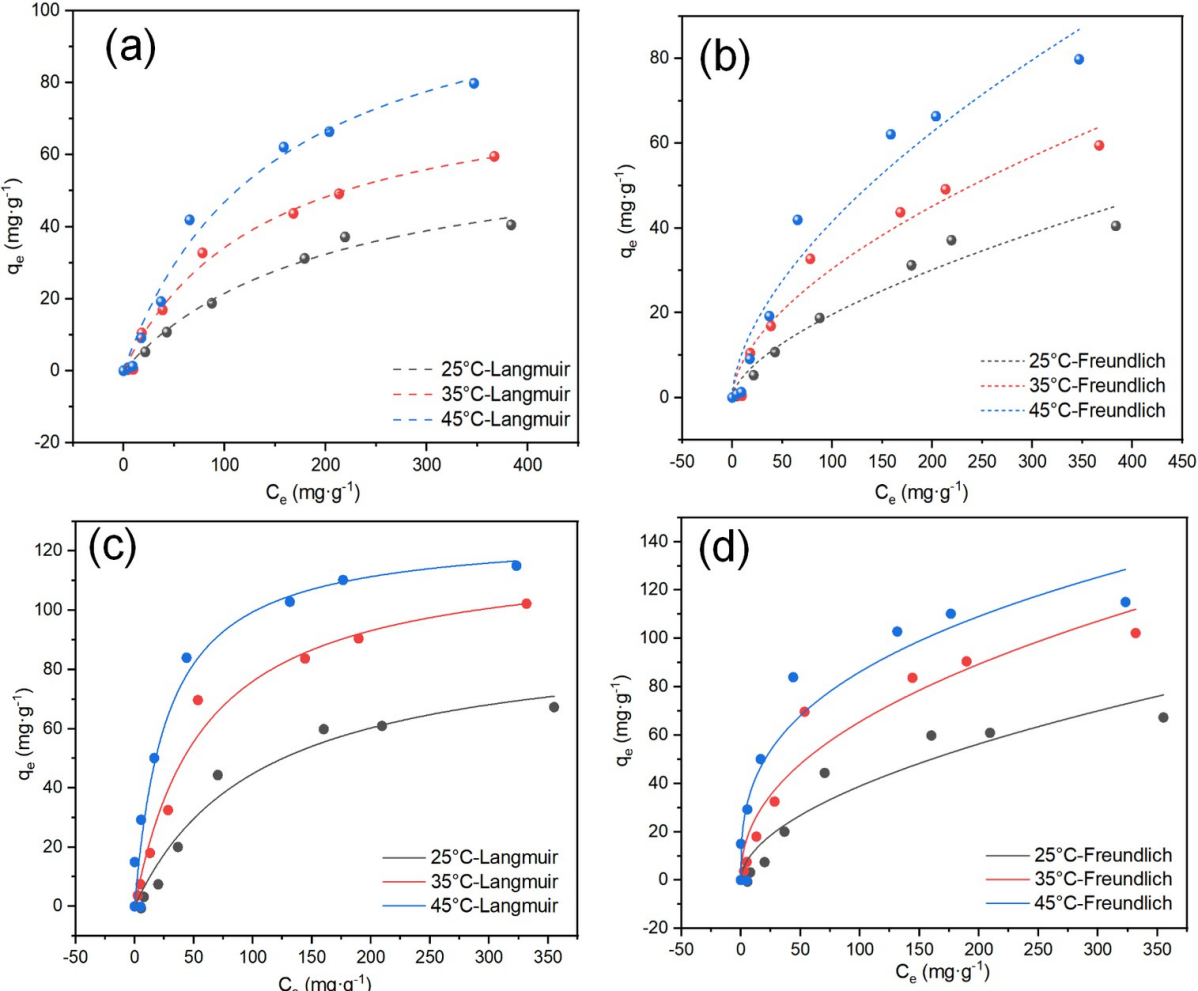
(a, b) NH_4_^+^–N and (c, d) PO_4_^3−^–P adsorption isotherms of MgSBC-0.5(@Al) at different temperatures.

The adsorption equilibrium isotherms of MgSBC-0.5(@Al) for NH_4_^+^–N and PO_4_^3−^–P were obtained at 298, 308, and 318 K. The adsorption process of NH_4_^+^–N may be accurately described by the Langmuir and Freundlich models (Fig 3A and 3B) (Table 4) (Eqs (3) and (4)). These results indicate that MgSBC-0.5(@Al) adsorbs NH_4_^+^–N through both monolayer and multilayer adsorption. The adsorbate was evenly distributed on the surface of the adsorbent, resulting in saturation [30]. The adsorption of PO_4_^3−^–P on MgSBC-0.5(@Al) at 298 K can be better described by the Langmuir model, as shown in Fig 3C and 3D. This suggests that the adsorption of PO_4_^3−^–P was likely monolayer. The Langmuir equation was used to determine the maximum adsorption capacities of MgSBC-0.5(@Al) for NH_4_^+^–N and PO_4_^3−^–P, yielding 65.19 and 92.10 mg·g^−1^, respectively, at 298 K. The maximum adsorption capacities of SBC for NH_4_^+^–N and PO_4_^3−^–P are 11.01 and 14.66 mg·g^−1^ (S2A and S2B Fig). At the same time, the *S*_BET_ of MgSBC-0.5(@Al) is 18.09 times larger than that of SBC. The higher adsorption capacity of MgSBC-0.5(@Al) could not be attributed solely to the chemical adsorption on MgO, AlOOH, Al_2_O_3_, and other Mg/Al/Si composites on the BC surface. Physical adsorption and other factors also likely contributed to it [31].

**Table 4:** Isotherm adsorption model parameters of MgSBC-0.5(@Al) and SBC at 298K.

Isotherm models	Parameters	Adsorbent-adsorbate			
		MgSBC-0.5(@Al)-a	MgSBC-0.5(@Al)-b	SBC-a	SBC-b
Langmuir	*Q* (mg·g^-1^)	65.19	92.10	11.01	14.66
	*K*_L_ (L·mg^-1^)	0.0049	0.0095	0.024	0.011
	*R* ^2^	0.987	0.967	0.925	0.992
Freundlich	*K*_F_	1.13	3.30	1.10	0.84
	1/n	0.62	0.54	0.39	0.46
	*R* ^2^	0.954	0.905	0.919	0.988

Notes: (“a”: the result of NH_4_^+^-N, and “b”: the result of PO_4_^3—^P).

NH_4_^+^–N and PO_4_^3−^–P adsorption capabilities of MgSBC-0.5(@Al) might be enhanced by increasing the reaction temperature. As presented in Table 5, with the increase in temperature from 298 to 318 K, the maximum adsorption capacities of NH_4_^+^–N and PO_4_^3−^–P increase from 65.19 to 115.28 mg·g^−1^, and from 92.10 to 126.49 mg·g^−1^, respectively.

**Table 5:** Isotherm adsorption model parameters of MgSBC-0.5(@Al) at 298, 308, and 318 K.

Models	Parameters	Values		
		298 K	308 K	318 K
Langmuir-a	*Q* (mg·g^-1^)	65.19	81.79	115.28
	*K*_L_ (L·mg^-1^)	0.0049	0.0072	0.0069
	R^2^	0.987	0.988	0.984
Freundlich-a	*K*_F_	1.13	2.20	2.71
	1/n	0.62	0.57	0.50
	*R* ^2^	0.954	0.958	0.948
Langmuir-b	*Q* (mg·g^-1^)	92.10	120.12	126.49
	*K*_L_ (L·mg^-1^)	0.0095	0.017	0.037
	R^2^	0.967	0.981	0.954
Freundlich-b	*K*_F_	3.30	8.36	17.91
	1/n	0.54	0.45	0.34
	*R* ^2^	0.905	0.930	0.889

Notes: (“a”: is the result of NH_4_^+^-N, and “b” is the result of PO_4_^3—^P).

The values of Δ*H* and Δ*G* were obtained from S2C Fig, indicating that the adsorption process is endothermic (Δ*H* > 0; Table 6) [32]. The value of Δ*G* is negative and increases in absolute value with increasing temperature. Thus, the adsorption occurred spontaneously, and an increase in temperature enhanced its efficiency. The Δ*S* > 0 indicates that disorder and degrees of freedom at the solid–liquid interface increase during adsorption. This increase in disorder favored the occurrence of chemical adsorption [33].

**Table 6:** Calculated thermodynamic parameters.

Pollutant	Temperature (k)	*K*_L_ (L·mg^-1^)	*K*_C_	Δ*H* (kJ·mol^-1^)	Δ*S* (kJ·mol^-1^·K^-1^)	Δ*G* (kJ·mol^-1^)
NH_4_^+^-N	298	0.0049	4929.51	12.73	0.11	-21.07
	308	0.00720	7189.28			-22.74
	318	0.0069	6849.32			-23.35
PO_4_^3—^P	298	0.0095	49907.02	52.60	0.27	-26.80
	308	0.017	90845.55			-29.24
	318	0.037	193772.20			-32.19

**Fig 4:**
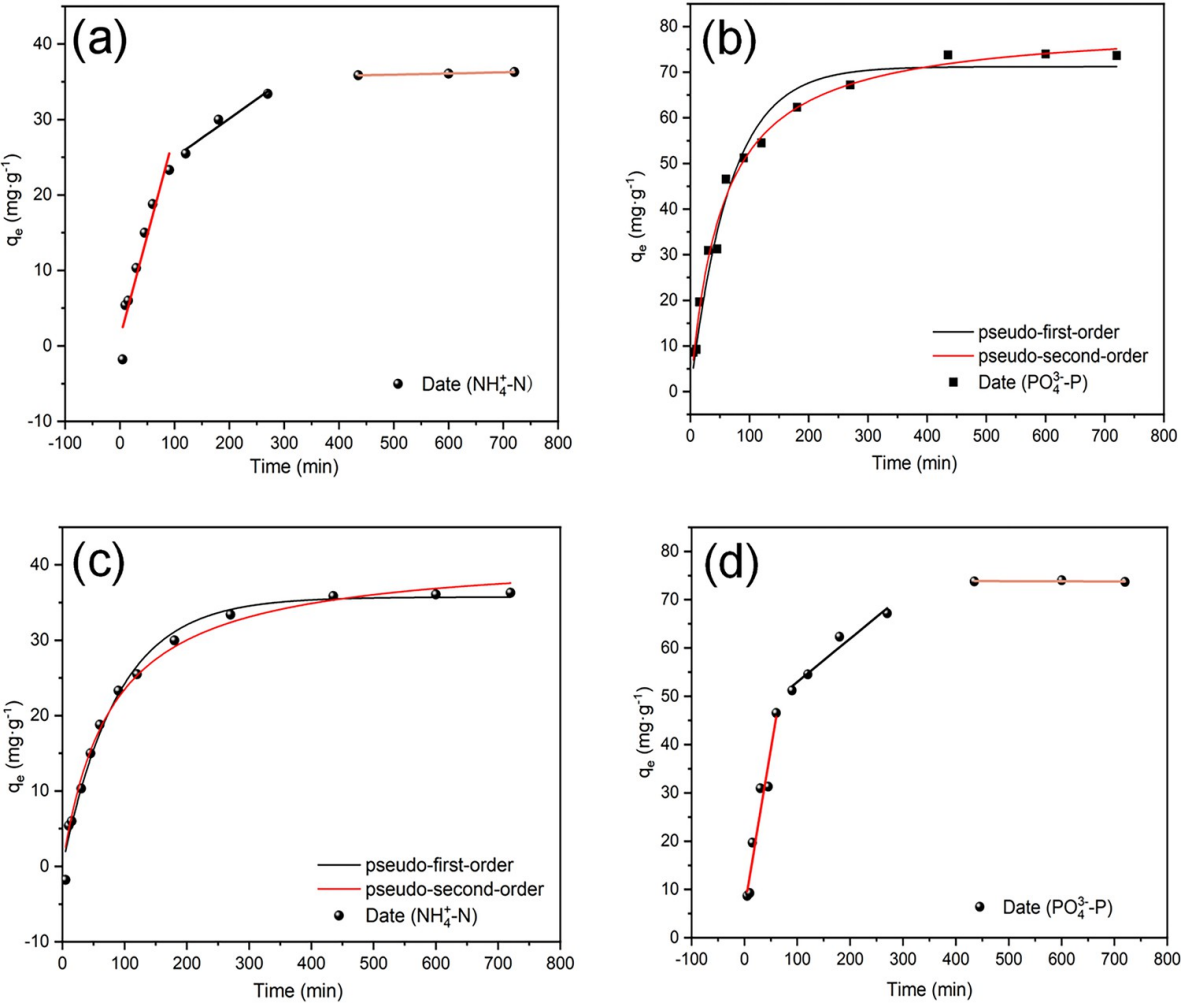
(a-b) The adsorption kinetics of MgSBC-0.5(@Al) for ammonia nitrogen: pseudo first-order and pseudo second-order models and Weber Morris models, and (c-d) pseudo first-order, pseudo second-order models and Weber Morris models for phosphate.

The adsorption of NH_4_^+^–N and PO_4_^3−^–P on MgSBC-0.5(@Al) was studied using pseudo-first-order and pseudo-second-order models. Fig 4A and 4B show the adsorption of NH_4_^+^–N and PO_4_^3−^–P on MgSBC-0.5(@Al) reaches equilibrium at around 90 and 270 min, respectively. Fitted with Eqs (5) and (6), the adsorption capacities of MgSBC-0.5(@Al) for NH_4_^+^–N and PO_4_^3−^–P are 33.40 and 67.21 mg·g^−1^, respectively. Table 7 shows that the R^2^ of the pseudo-second-order kinetic model (0.988) is higher than that of the pseudo-first-order kinetic model (0.973) for PO_4_^3−^–P adsorption. This implies that PO_4_^3−^–P adsorption was primarily a chemical process. This mechanism involved multiple components, including the valence force, the exchange or sharing of electrons between the adsorbent and the adsorbate, and the potential synthesis of new compounds. At the same time, the R^2^ of the pseudo-first-order kinetic model (0.988) is higher than that of the pseudo-second-order kinetic model (0.986) for the adsorption of NH_4_^+^–N. This suggests that NH_4_^+^–N was adsorbed primarily through physical adsorption [34]. However, the diffusion mechanism of the adsorbent could not be accurately identified based on the pseudo-first-order and pseudo-second-order models. Therefore, the Weber–Morris equation was employed to comprehensively investigate the rate-limiting mechanism of the adsorption process [35].

**Table 7:** Adsorption kinetics parameters of MgSBC-0.5(@Al) adsorption of NH4+-N and PO43—P.

Isotherm models	Parameters	Adsorbent-adsorbate	
		MgSBC-0.5(@Al)- NH_4_^+^-N	MgSBC-0.5(@Al)- PO_4_^3—^P
Pseudo-first-order	*Q*_e_ (mg·g^-1^)	35.73	71.21
	*k*_1_ (min^-1^)	0.01137	0.015
	*R* ^2^	0.988	0.973
Pseudo-second-order	*Q*_e_ (mg·g^-1^)	41.60	80.68
	*k*_2_ (g (mg·min) ^-1^)	3.1175912×10^−4^	2.3189232×10^−4^
	*R* ^2^	0.98578986	0.98801
Weber-Morris	*k*_*i2*_ (mg g^-1^ min^-0.5^)	0.05152	0.08979
	*R* ^2^	0.92805	0.93755938

If adsorption was constrained solely by intra-particle diffusion, the regression line would pass through the origin. However, the intercept (*C*) of the NH_4_^+^–N and PO_4_^3−^–P adsorption curve is not equal to zero (Fig 4C and 4D) (Eq (7)), suggesting that intra-particle diffusion is not the sole governing force of adsorption [36]. The adsorption of NH_4_^+^–N and PO_4_^3−^–P exhibits a multilinear pattern. The first curve in the graph shows the fast adsorption of contaminants over numerous binding sites within a brief timeframe, accompanied by cation exchange for NH_4_^+^–N. The second curve indicates that diffusion within the particles played a major role, and that NH_4_^+^–N and PO_4_^3−^–P diffused into the particles. The last curve exhibits a decrease in the rate of diffusion within the particles because of the lower pollutant concentrations in the solution. The second-stage curves for NH_4_^+^–N and PO_4_^3−^–P exhibit higher R^2^ values (0.99 and 0.96, respectively). The Weber–Morris model was used to explain the adsorption of NH_4_^+^–N and PO_4_^3−^–P on BC. This model suggests that diffusion within the particles played a major role in adsorption. However, other mechanisms also limited the rate of adsorption. The adsorption of NH_4_^+^–N and PO_4_^3−^–P was likely controlled by several factors, such as rapid adsorption on the outer surface, ion exchange between Mg/Al, and simultaneous diffusion inside the SBC [37,38].

### Effects of different factors on NH_4_^+^–N and PO_4_^3−^–P capture

**Fig 5:**
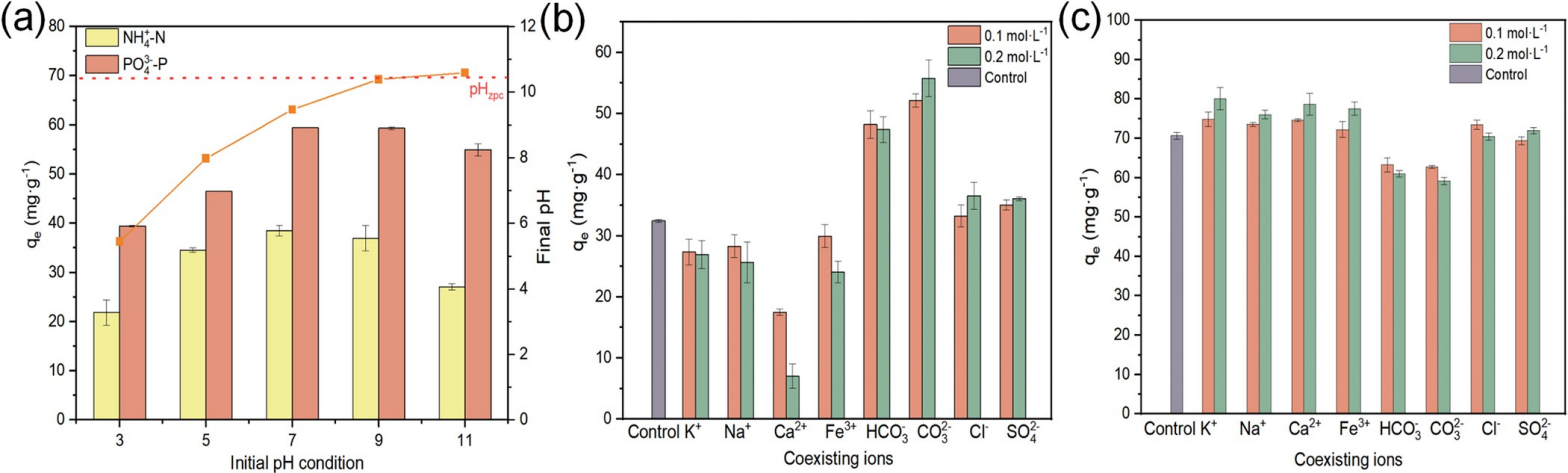
(a) Effect of initial pH of solution on adsorption of ammonia nitrogen and phosphorus by MgSBC-0.5(@Al), (b-c) The effect of coexisting ions on the removal of ammonia nitrogen and phosphorus. (t = 35°C, pH = 7, adsorbent dosage 0.67 g·L^-1^).

With the increase in pH from 3 to 7, the adsorption capacity for NH_4_^+^–N gradually increases, reaching a maximum of 38.48 mg·g^−1^ (Fig 5A). With the increase in pH from 7 to 9, no changes are observed. At the same time, at pH > 9, the adsorption capacity for NH_4_^+^–N decreases. For PO_4_^3−^–P, the adsorption capacity gradually increases with pH, from 3 to 7, reaching 59.44 mg·g^−1^. At pH > 7, the adsorption capacity for PO_4_^3−^–P decreases. At the same time, at pH < 3, the adsorption capacity of MgSBC-0.5(@Al) for NH_4_^+^–N and PO_4_^3−^–P significantly decreases. At pH > 9, the adsorption capacities for NH_4_^+^–N and PO_4_^3−^–P also notably decrease. Such results were attributed to (1) the reduction of dissociated carboxyl groups in BC at low pH [39], causing a relatively low adsorption capacity for PO_4_^3−^–P. (2) PO_4_^3−^–P existed in different forms at different pH: H_2_PO_4_^−^ (pk_1_ = 2.15) at pH < 7.20, HPO_4_^2−^ (pk_2_ = 7.20) at pH of 7.20–12.33, and PO_4_^3−^ at pH > 12.33. The lower adsorption free energy of H_2_PO_4_^−^ compared to HPO_4_^2−^ indicates that the former would be more readily adsorbed by the adsorbent. Consequently, the adsorption of PO_4_^3−^–P was strongly affected by pH in the range of 7.20–12.22 [40,41]. (3) The zero point potential (pH_ZPC_) of MgSBC-0.5(@Al) was 10.45, indicating the presence of hydroxyl ligands on its surface, which facilitated the adsorption of PO_4_^3—^P. When the initial pH>pHzpc, the surface of MgSBC-0.5(@Al) carried a negative charge. There was a repulsive force between PO_4_^3—^P and MgSBC-0.5(@Al), which inhibited electrostatic adsorption. However, in this case, chemical interactions such as precipitation reactions between Mg^2+^and HPO_4_^2−^ might contribute to the removal of PO_4_^3—^P [42,43]. (4) Electrostatic repulsion between negatively charged PO_4_^3−^–P anions and the deprotonated surface of BC strengthened with the increase in the concentration of OH^−^ in the solution at pH of 9–11. When the pH rose to about 11, a high pH corresponded to an increase in the concentration of hydroxide ions (OH^−^) in the solution. NH_4_^+^-N was mainly in the form of NH_3_·H_2_O. Therefore, the adsorption capacity of MgSBC-0.5(@Al) for NH_4_^+^-N was weakened. So the overall adsorption effect was decreased. [29,44]. At equilibrium, the pH is maintained at 8–10, which was in the pH zone conducive to struvite crystallization (pH > 8.5). This indicated that the modification of SBC considerably increased its adsorption for NH_4_^+^–N and PO_4_^3−^–P.

The effect of co-presence of several cations and anions, such as K^+^, Ca^2+^, Fe_3_^+^, Na^+^, SO_4_^2−^, Cl^−^, HCO_3_^−^, and CO_3_^2−^, in wastewater on the removal of NH_4_^+^–N and PO_4_^3−^–P by MgSBC-0.5(@Al) was investigated. The results of the interference test for single coexisting ions are shown in Fig 5B and 5c. K^+^ and Na^+^ in the solution competed with NH_4_^+^–N and reacted with PO_4_^3−^–P to form KMgPO_4_·7H_2_O and NaMgPO_4_·7H_2_O. Ca^2+^ and Fe^3+^ in the solution would immediately react with PO_4_^3−^–P to form amorphous CaCO_3_ and FePO_4_, respectively, producing irregular crystals and seriously hindering the crystallization of struvite. Hence, the presence of K^+^, Na^+^, Ca^2+^, and Fe^3+^ in the solution led to an increase in PO_4_^3−^–P adsorption capacity and a decrease in NH_4_^+^–N adsorption capacity [45–47]. SO_4_^2−^ and Cl^−^ show negligible effects on adsorption. However, the presence of HCO_3_^–^ or CO_3_^2−^ promoted the adsorption of NH_4_^+^–N by MgSBC-0.5(@Al). One potential explanation is that the presence of these two ions might have resulted in a pH increase, strengthening electrostatic attraction between NH_4_^+^–N and the adsorbent, thus enhancing the adsorption efficiency. At the same time, the adsorption capacity for PO_4_^3−^–P decreased. This likely occurred because carbonate inhibited the precipitation of struvite, resulting in a pH increase and a consequent increase in the electrostatic repulsion between PO_4_^3−^–P and the adsorbent [48].

To study the N and P removal performance of MgSBC-0.5(@Al) on real samples, adsorption experiments were conducted using anaerobic-fermentation biogas slurry. The total phosphorus and total nitrogen concentrations in the biogas slurry were 31.4 ± 1.0 and 50.1 mg·L^−1^, respectively, and its pH was 7.7 ± 0.2. As shown in S3 Fig, with the increase in solid–liquid ratio from 0.33 to 6.70 g·L^−1^, the removal rates of total phosphorus and total nitrogen increase. The removal rates reach 71.34% and 69.69%, respectively, and then stabilize. At the same time, SBC at a dosage of 6.7 g·L^−1^ shows the total nitrogen and total phosphorus removal rates of only 10.06% and 1.03%, respectively. This indicated that for MgSBC-0.5(@Al), 6.70 g·L^−1^ was the optimal solid–liquid ratio for treating actual biogas slurry, and modified sludge-based biochar had a better treatment effect on actual biogas slurry. But compared to the simulated solution, the adsorbent had a poor effect on nitrogen and phosphorus in the biogas slurry. This was attributed to the presence of substances in the biogas slurry competing with nitrogen and phosphorus for adsorption sites on MgSBC-0.5(@Al), resulting in decreased adsorption capacity.

### Mechanism of NH_4_^+^–N and PO_4_^3−^–P capture

**Fig 6:**
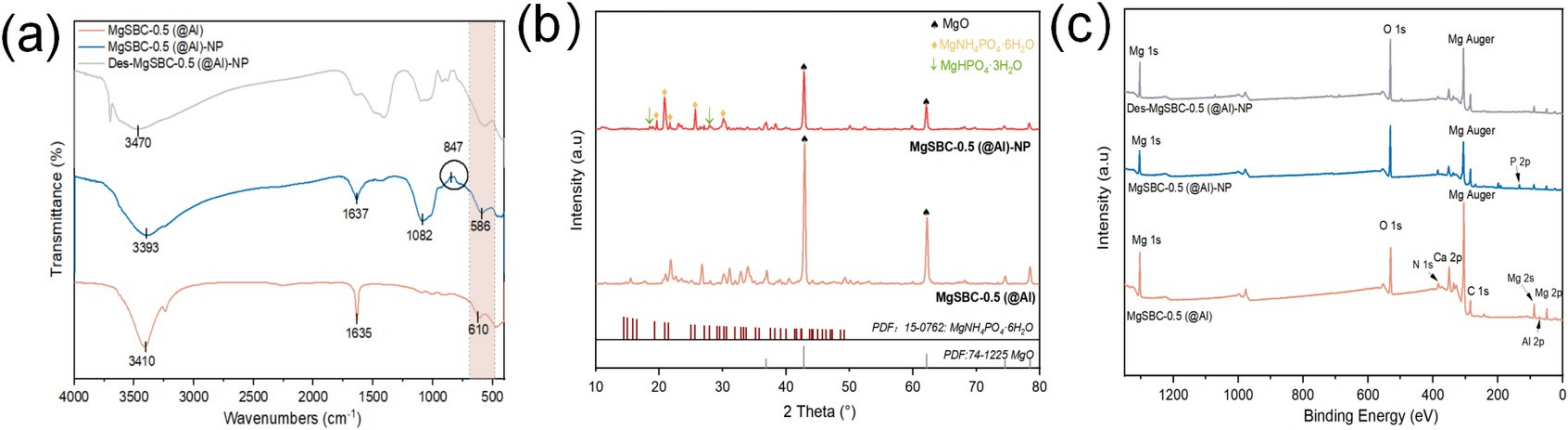
(a-c) FTIR, XRD and XPS spectra of MgSBC-0.5(@Al) adsorption of NH_4_^+—^N and PO_4_^3—^P.

To validate the adsorption mechanism, further characterization was conducted through FTIR and XPS. Fig 6A shows the FTIR spectrum. The bound water–OH vibration peak of MgSBC-0.5(@Al) shifts from 3410 to 3393 cm^−1^ after adsorption and then to 3470 cm^−1^ after desorption (Fig 6A). This transition suggests that adsorption–desorption involves the formation and breakage of hydrogen bonds and electrostatic attraction between pollutants and the adsorbent [49]. The peaks at 586 and 610 cm^−1^ remained within the range of metal–oxygen bonding (400–650 cm^−1^). In particular, MgSBC-0.5(@Al)–NP shows a peak near the 847 cm^−1^, which was attributed to the deformation of–OH linked to Mg^2+^ produced by Mg(OH)_2_. The observed shift in the peak around the wavelength 1636 cm^−1^ was correspond to the adsorption of NH_4_^+^–N, resulting in a widening of the peak. Additionally, the–OH vibration peak transformed into a symmetric bending vibration peak of N–H in NH_4_ units after adsorption. Furthermore, the peak at 1082 cm^−1^ was assigned to the asymmetric stretching vibration of P–O. Considering crystal structure, struvite consists of three distinct functional groups: (1) PO_4_ tetrahedron, (2) Mg·6H_2_O octahedron, and (3) NH_4_ groups. These groups are connected by hydrogen bonds. The presence of NH_4_^+^–N and PO_4_^3−^–P adsorbed on the BC was confirmed through FTIR [50].

The XRD images of MgSBC-0.5(@Al) before and after NH_4_^+^-N and PO_4_^3—^P adsorption are compared to further elucidated the adsorption mechanism (Fig 6B). It could see that the MgO peak in the XRD spectrum of biochar is weakened. A series of XRD peaks appeared at 20.85° (111), 21.45° (021), 25.72° (200) and 30.19° (012) for MgSBC-0.5(@Al), corresponding to the MgNH_4_PO_4`_H_2_O diffraction peak. This indicated that the synthesis of struvite crystallization.

**Fig 7:**
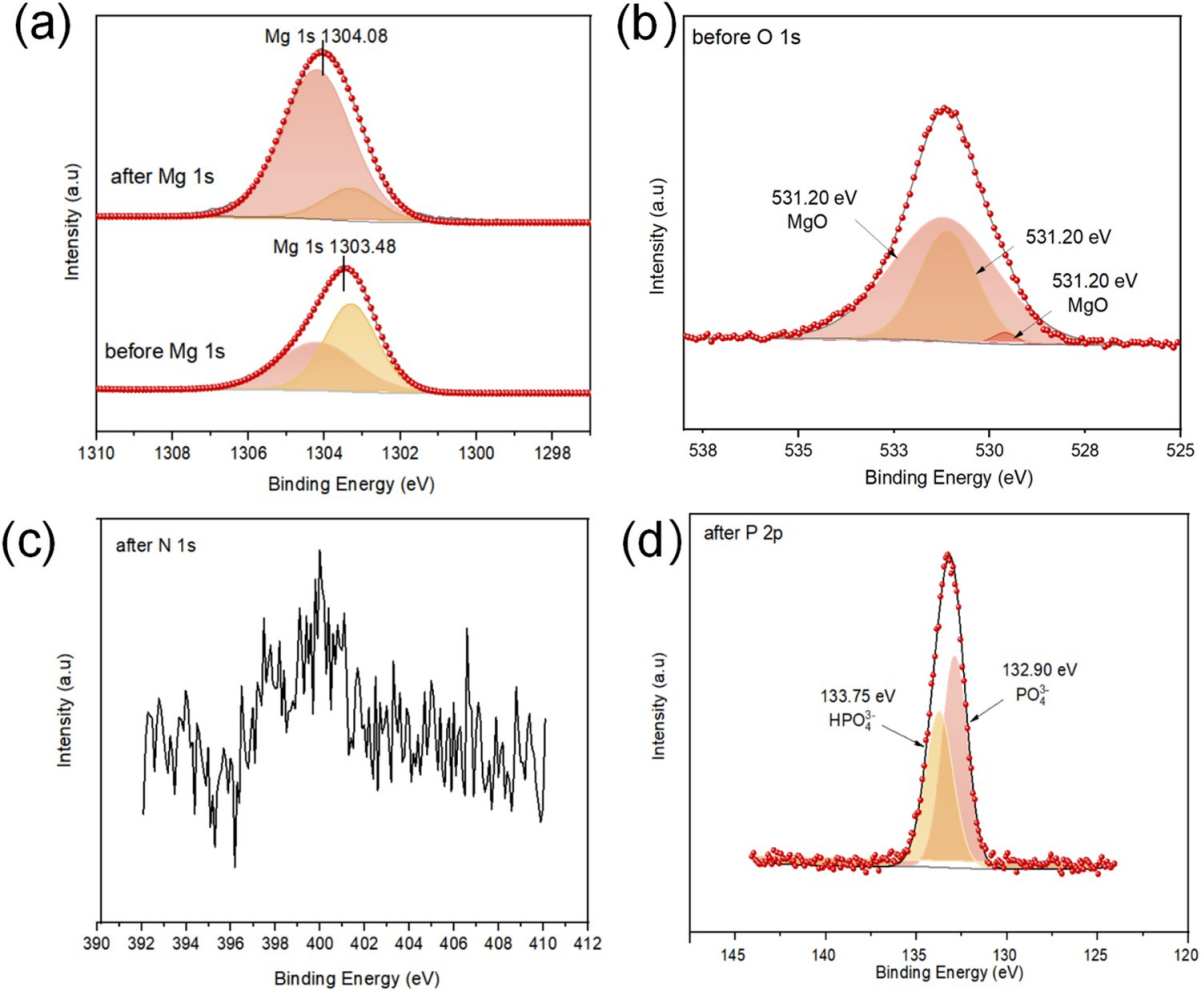
(a) XPS spectra of Mg 1s, (b) XPS spectra of O 1s before utilization, and (c-d) XPS spectra of N 1s and P 2p after utilization.

XPS was used to analyze the changes in the chemical composition of MgSBC-0.5(@Al) caused by adsorption. Fig 6C shows that the N 1s and P 2p characteristic peaks of MgSBC-0.5(@Al)–NP are notably more prominent than those of MgSBC-0.5(@Al) and Des-MgSBC-0.5(@Al)–NP. This suggests that NH_4_^+^–N and PO_4_^3−^–P were adsorbed. Additionally, the contents of trace elements (Mg, Ca, Al, and Fe) decreased after adsorption, as indicated in Table 8 [51]. As shown in Fig 7A, the Mg 1s peak shifted from 1303.48 to 1304.08 eV after adsorption, indicating a change in the chemical state of Mg. The O 1s peak is composed of Mg–O (531.2 and 529.6 eV) and Al(OH)_3_ (or Mg/Al composite; 531.10 eV) peaks (Fig 7B) [52,53]. After the adsorption of NH_4_^+^–N, a weak peak corresponding to the binding energy of N 1s at 400.01 eV appears in the XPS spectrum, proving that NH_4_^+^ ions were successfully adsorbed on the BC (Fig 7C). The P 2p peaks at 132.9 and 133.75 eV correspond to HPO_4_^3−^ and PO_4_^3−^, respectively (Fig 7D) [54–56]. Although the solubility of MgO and Al_2_O_3_ is very low, the introduction of PO_4_^3−^–P anion into the solution promoted their dissolution, resulting in the formation of greater amounts of insoluble salts (MgHPO_4_ and MgNH_4_PO_4_·6H_2_O). Therefore, the adsorption on MgSBC-0.5(@Al) likely involved precipitation reactions.

**Table 8:** Element composition (atomic%) of MgSBC-0.5(@Al) and MgSBC-0.5(@Al)-NP measured by XPS and XRF.

Analysis methods	Sample	C	O	Si	Mg	Al	Ca	Fe	P	N	Other elements
XPS	MgSBC-0.5(@Al)	14.34	23.69	0.43	28.08	6.14	4.56	9.23	2.01	0.49	/
	MgSBC-0.5(@Al)-NP	8.97	24.73	1.68	20.57	3.64	1.36	6.05	26.91	5.09	/
XRF	MgSBC-0.5(@Al)-NP	6.90	27.26	7.14	16.18	3.97	0.93	7.62	14.65	10.32	5.03

The XRF spectrum of the entire MgSBC-0.5(@Al)–NP sample (Table 9) shows the presence of insoluble mineral phases on the surface and in the bulk of MgSBC-0.5(@Al). The most abundant inorganic component of MgSBC-0.5(@Al)–NP was Mg; additionally, the sample contains N (10.32%) and P (14.65%), and the N content was considerably higher than that detected using XPS. It might be that only a part of the NH_4_^+^–N was adsorbed on the material surface, and the other part physically adsorbed in the pores of the biochar, which was consistent with the conclusion of the kinetic model [57]. The removal of NH_4_^+^–N was mainly controlled by physical adsorption. The Al, Ca, and Fe contents of MgSBC-0.5(@Al)–NP are consistent with the XPS results, indicating that most of these metal oxides were loaded on the surface of the material. Silicon accounts for 7.14%, indicating that Si was distributed inside the material. The overall spectrum is shown in S4 Fig.

**Table 9:** Effects of five groups on yield and characters of mung bean sprouts.

Group	Stem Length (cm)	Root Length (cm)	Average wet weight (g)	Average dry weight (g)	Nitrogen (g·kg^-1^)	Phosphorus (g·kg^-1^)
A	12.58 ± 3.33b	9.15 ± 4.10ab	0.42 ± 0.041b	0.085 ± 0.0068b	41.28	3.86
B	11.52 ± 2.52c	8.21 ± 3.17ab	0.36 ± 0.029c	0.069 ± 0.0089c	30.07	2.21
C	8.92 ± 3.89c	7.02 ± 2.54b	0.32 ± 0.024d	0.066 ± 0.0038cd	29.12	2.16
D	16.79 ± 1.41c	9.34 ± 2.32a	0.65 ± 0.035a	0.092 ± 0.0080a	57.17	5.96
E	8.58 ± 0.92a	7.99 ± 1.38 ab	0.34 ± 0.033c	0.063 ± 0.0048d	29.66	2.17

Note: 1: Repeat 4 times for each group of samples; 2: Values with superscript letters a, b, c and d are significantly different across columns (p<005).

The adsorption mechanisms were inferred based on the physical and chemical properties of MgSBC-0.5(@Al), *S*_BET_, XRD, FTIR, XPS, and other relevant data. The adsorption mechanism was concluded to include the following phenomena. (1) The physical adsorption capacity of biochar was enhanced through electro-assisted modification with Mg and thermal modification, resulting in a considerable increase in the surface area of MgSBC-0.5(@Al). These processes also increased the number of adsorption sites on the material, improving its physical adsorption capacity. (2) The reduction of MgO and Al_2_O_3_ to Mg(OH)_2_ and Al(OH)_3_ at the solid–liquid interface caused an increase in pH. At pH of the aqueous solution above 2.15, H_2_PO_4_^−^, HPO_4_^2−^, and PO_4_^3−^ were the main forms of PO_4_^3−^–P. Pollutants were removed by electrostatic adsorption [58]. (3) Struvite crystallization was the primary mechanism of the removal of NH_4_^+^–N and PO_4_^3−^–P. At the solid–liquid interface, MgO formed a hydrated intermediate (Mg(OH)_2_), which reacted with NH_4_^+^–N and PO_4_^3−^–P in the solution to form MgNH_4_PO_4_·6H_2_O. The main reactions are shown in Eqs (12–18).


$$MgO+H_{2}O\to Mg^{2+}+2OH^{-}$$ (13)

$$MgO+H_{2}O\to MgOH^{+}+OH^{-}$$ (14)

$$MgOH^{+}+HPO_{4}^{2-}\to MgOH^{+}\cdots HPO_{4}^{2-}$$ (15)

$$Mg^{2+}+HPO_{4}^{2‐}\to MgHPO_{4}$$ (16)

$$Mg^{2+}+NH_{4}^{+}+H_{2}PO_{4}^{‐}+6H_{2}O=MgNH_{4}PO_{4}\cdot 6H_{2}O↓+2H^{+}$$ (17)

$$Mg^{2+}+NH_{4}^{+}+HPO_{4}^{2‐}+6H_{2}O=MgNH_{4}PO_{4}\cdot 6H_{2}O↓+H^{+}$$ (18)

$$Mg^{2+}+NH_{4}^{+}+PO_{4}^{3‐}+6H_{2}O=MgNH_{4}PO_{4}\cdot 6H_{2}O↓$$ (19)

### Nitrogen and phosphorus recovery

**Fig 8:**
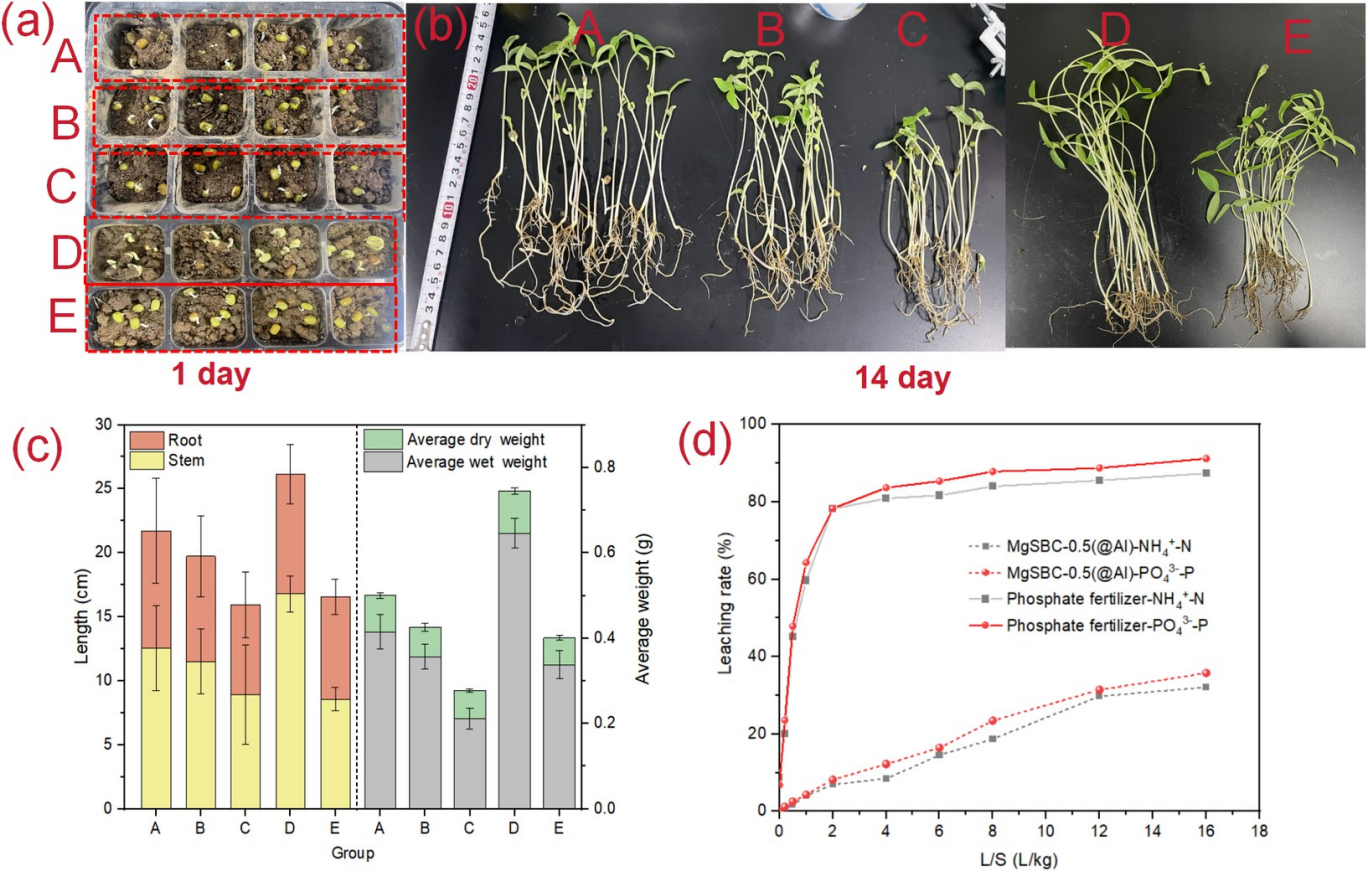
(a-b) Growth of mung beans on days 1 and 14, (c) Root length, stem length, average wet weight and average dry weight of five groups of mung beans on day 14, and (d) NH_4_^+^-N and PO_4_^3—^P leaching rates of MgSBC-0.5(@Al)-NP and commercial fertilizers.

Pot experiments were employed to test the applicability of MgSBC-0.5(@Al)–NP as an N and P fertilizer for plant growth. The germination rates of mung bean seeds in the five groups were 95%, 90%, 95%,90% and 85%, respectively, indicating that the addition of BC had little effect on germination rate. The photos of the plants at days 1, and 14 are shown in Fig 8A and 8B. However, as shown in Fig 8C, on day 14, mung bean sprouts in group A are on average substantially larger than those of group B, group C and group (Fig 8C). But it was lower than that group D. The average wet weight and dry weight of seedlings in group A were 0.42 g and 0,088 g (Table 9). After calculation, for stem length, average wet weight and average dry weight, there were significantly differences between groups A and E. And Group A was significantly different from the other three groups (Groups B, C, and D) (p<005). But for Root length. There was only a significantly difference between groups C and D. Although the growth and development of bean sprouts in group D (with the addition of commercial fertilizer) was slightly higher than that of group A, but the growth and development of mung bean sprouts in group A was much better than that of groups B, C and E. The contents of nitrogen and phosphorus in mung bean sprouts in group A (41.28 mg·kg^-1^ and 3.86 mg·kg^-1)^ were also higher than those in groups B, C and D. The growth and development of mung bean sprouts in group A was promoted by additional N and P in the soil. Notably, Mg-loaded BC had also been reported to promote crop growth [59], in part because its stable carbon structure improved soil microbial activity [60]. Therefore, MgSBC-0.5(@Al)–NP could be used as an N and P fertilizer.

To investigate the sustained release effect of MgSBC-0.5(@Al)–NP on NH_4_^+^–N and PO_4_^3−^–P after adsorption, leaching experiments were conducted. The leaching results of NH_4_^+^–N and PO_4_^3−^–P are shown in Fig 8D. The leaching characteristics of MgSBC-0.5(@Al)–NP were different from those of commercial fertilizers. The release of NH_4_^+^–N and PO_4_^3−^–P in MgSBC-0.5(@Al)-NP showed a gradually increasing trend through the leaching process. When the L/S ratio reached 2.0 L·kg^-1^, the cumulative leaching amounts of NH_4_^+^–N and PO_4_^3−^–P were only 2.53 mg·g^-1^(6.98%) and 6.05 mg/g (8.21%). At the end of the experiment, the cumulative leaching amounts of NH_4_^+^–N and PO_4_^3−^–P were 11.54 mg·g^-1^(32.12%) and 26.36 mg·g^-1^ (35.78%), which were 2.72 times and 2.54 times lower than those of commercial fertilizers, respectively. The generated MgHPO_4_·3H_2_O and MgNH_4_PO_4_·6H_2_O reduced the leaching of NH_4_^+^–N and PO_4_^3−^–P, forming a sustained release effect of MgSBC-0.5(@Al)-NP [61].

## Conclusions

Mg/Al-modified SBC was prepared via the electro-assisted modification of sludge followed by pyrolysis. The specific surface area of MgSBC-0.5(@Al) was 11.27 times higher than that of SBC and 18.06 times higher than that of MgSBC-0.5. The surface of biochar was covered with oxygen-containing metal complexes, including MgO, AlOOH, and Al_2_O_3_. The maximum adsorption capacities of MgSBC-0.5(@Al) for NH_4_^+^–N and PO_4_^3−^–P were 65.19 and 92.10 mg·g^−1^, higher by 4.45 and 6.28 times than those of SBC, respectively. The adsorption experiments and material characterization results confirmed that adsorption occurred through physical adsorption, electrostatic attraction, and struvite crystallization. The higher adsorption capacity of the Mg/Al-modified SBC was attributed to the transformation of the main adsorption form from physical adsorption to chemical adsorption. Furthermore, saturated MgSBC-0.5(@Al) was found to be a promising soil amendment. Therefore, electro-assisted modification showed substantial potential in the synthesis of Mg/Al bimetallic-modified BC and effectively increased the adsorption capabilities of SBC toward NH_4_^+^–N and PO_4_^3−^–P.

## Supporting information

S1 Fig (a) The effect of Mg-loading for BC on the adsorption of NH_4_^+^-N and PO_4_^3^-P and (b) the XRD patterns of MgSBC-0.1(@Al), MgSBC-0.25 (@Al), MgSBC-0.5(@Al), and MgSBC-1(@Al).
(TIF)

S2 Fig (a) The fitting of adsorption isotherms of NH_4_^+^-N and PO_4_^3^-P by SBC at 298K and (b) adsorption kinetics of NH_4_^+^-N and PO_4_^3^-P by SBC: pseudo first-order and pseudo second-order model fitting, and (c-d) The effect of temperature on the adsorption capacity of NH_4_^+^-N and PO_4_^3^-P.
(TIF)

S3 Fig The removal efficiency of MgSBC-0.5(@Al) on total nitrogen and total phosphorus in actual biogas slurry. (TIF)

S4 Fig XRF spectrum of MgSBC-0.5(@Al)-NP. (TIF)

S1 Graphical abstract (TIF)
