# Supplementary figures and images for: Simultaneous adsorption of ammonia nitrogen and phosphate on electro-assisted magnesium/aluminum-loaded sludge-based biochar and its utilization as a plant fertilizer
Source: PLoS One. 2024 Oct 25;19(10):e0311430. doi: 10.1371/journal.pone.0311430 (PMC11508052; doi:10.1371/journal.pone.0311430)

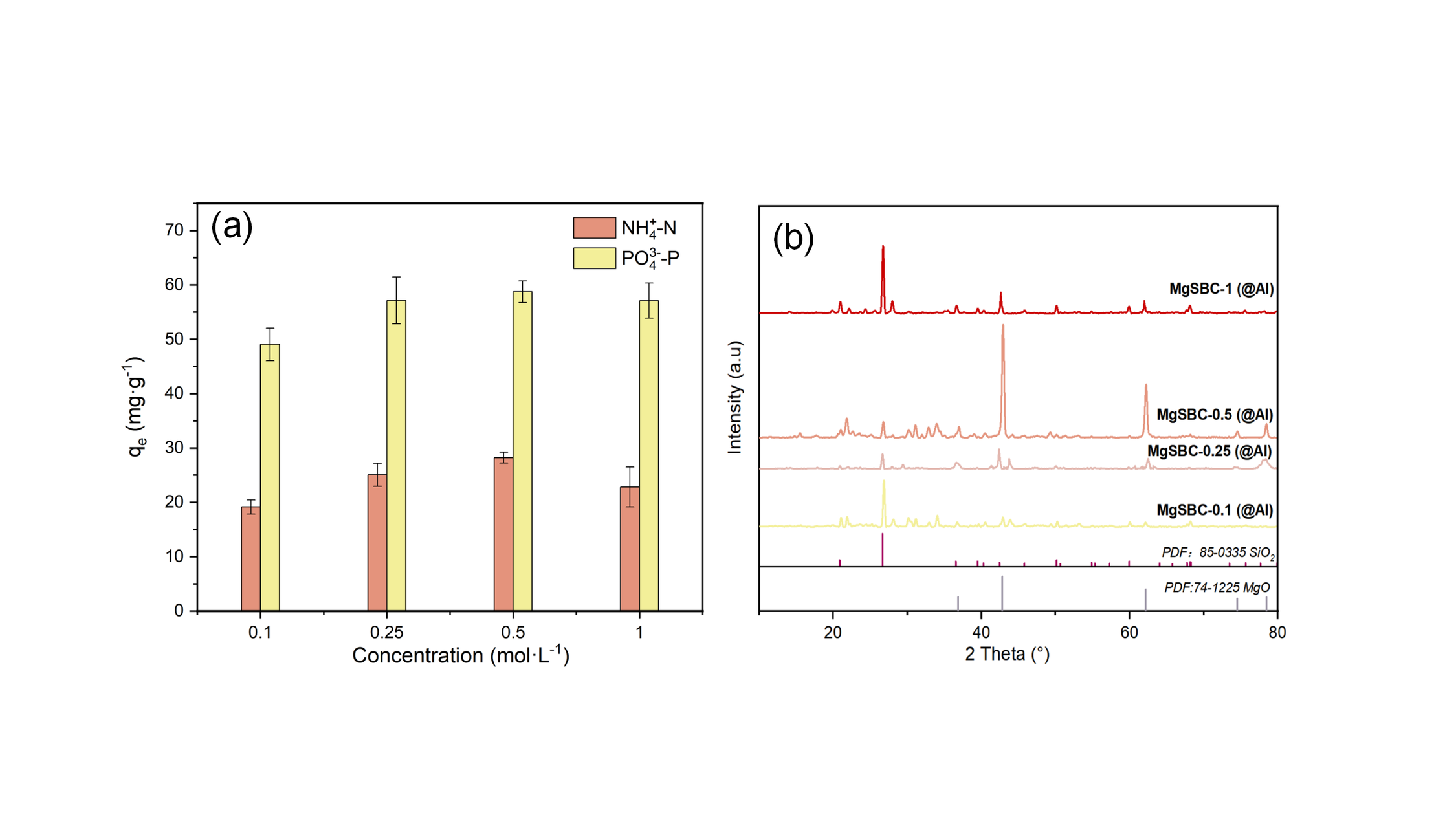

Supplement: S1 Fig — (a) The effect of Mg-loading for BC on the adsorption of NH4+-N and PO43-P and (b) the XRD patterns of MgSBC-0.1(@Al), MgSBC-0.25 (@Al), MgSBC-0.5(@Al), and MgSBC-1(@Al). (TIF) [file pone.0311430.s001.tif]

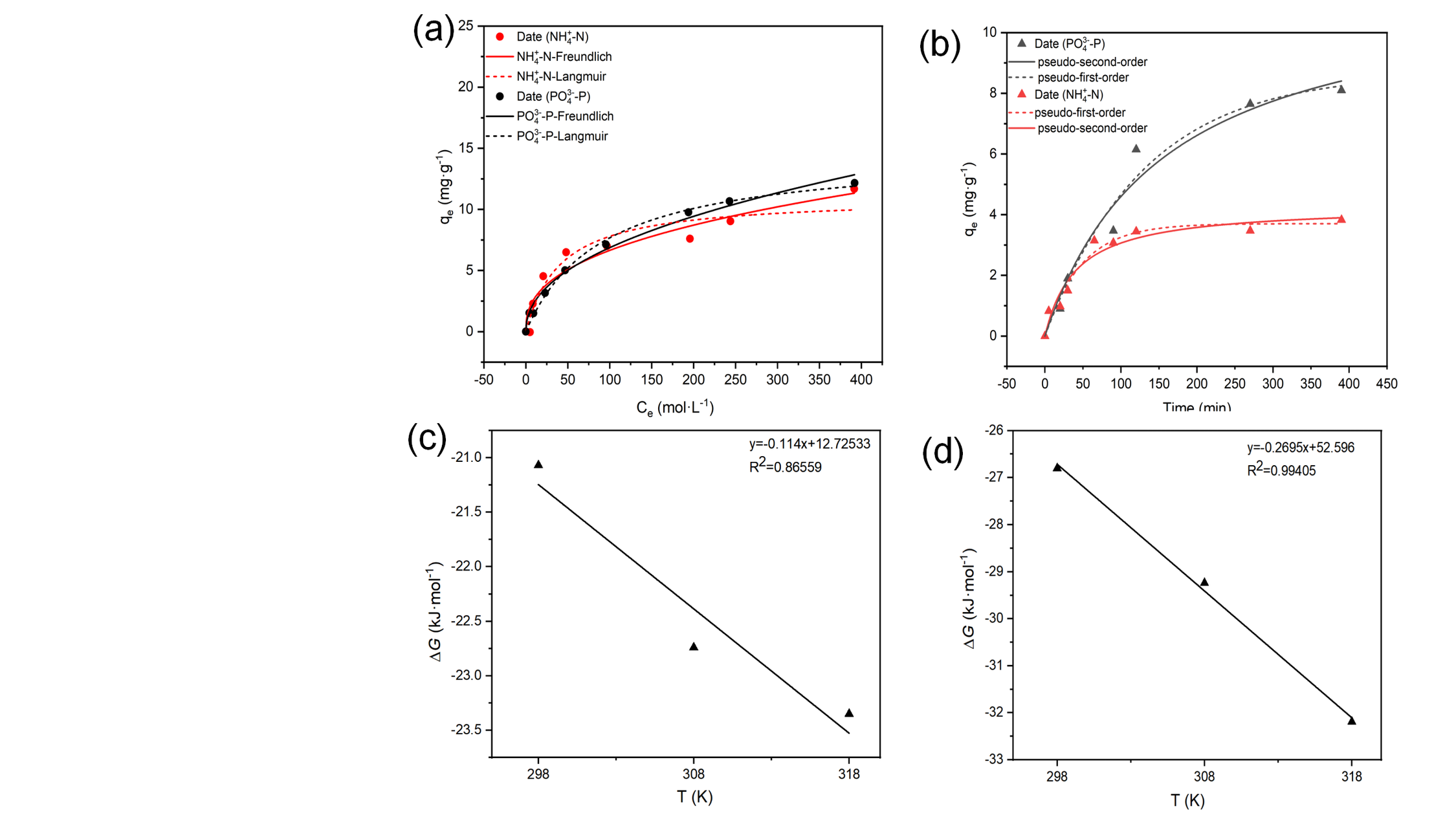

Supplement: S2 Fig — (a) The fitting of adsorption isotherms of NH4+-N and PO43-P by SBC at 298K and (b) adsorption kinetics of NH4+-N and PO43-P by SBC: pseudo first-order and pseudo second-order model fitting, and (c-d) The effect of temperature on the adsorption capacity of NH4+-N and PO43-P. (TIF) [file pone.0311430.s002.tif]

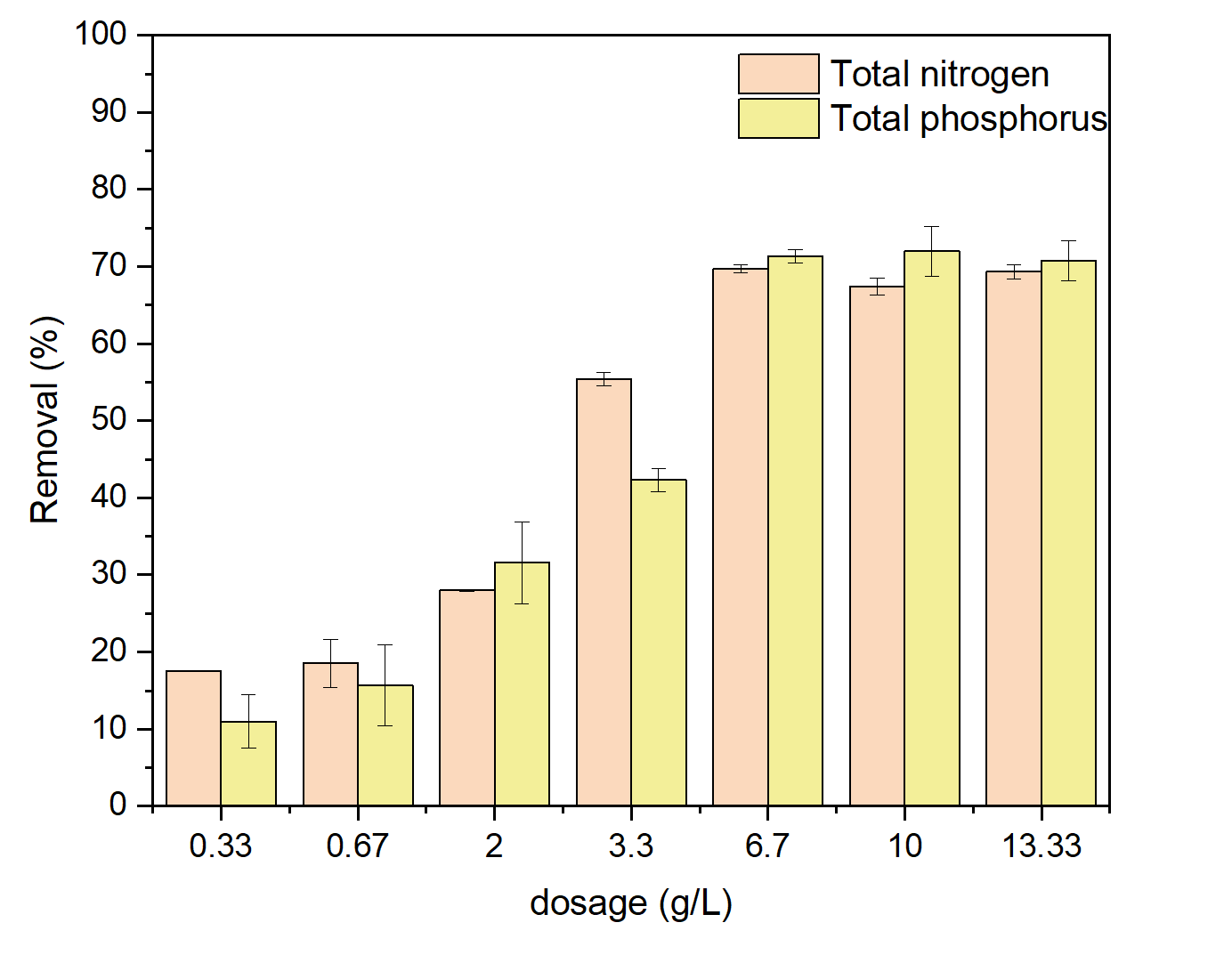

Supplement: S3 Fig — (TIF) [file pone.0311430.s003.tif]

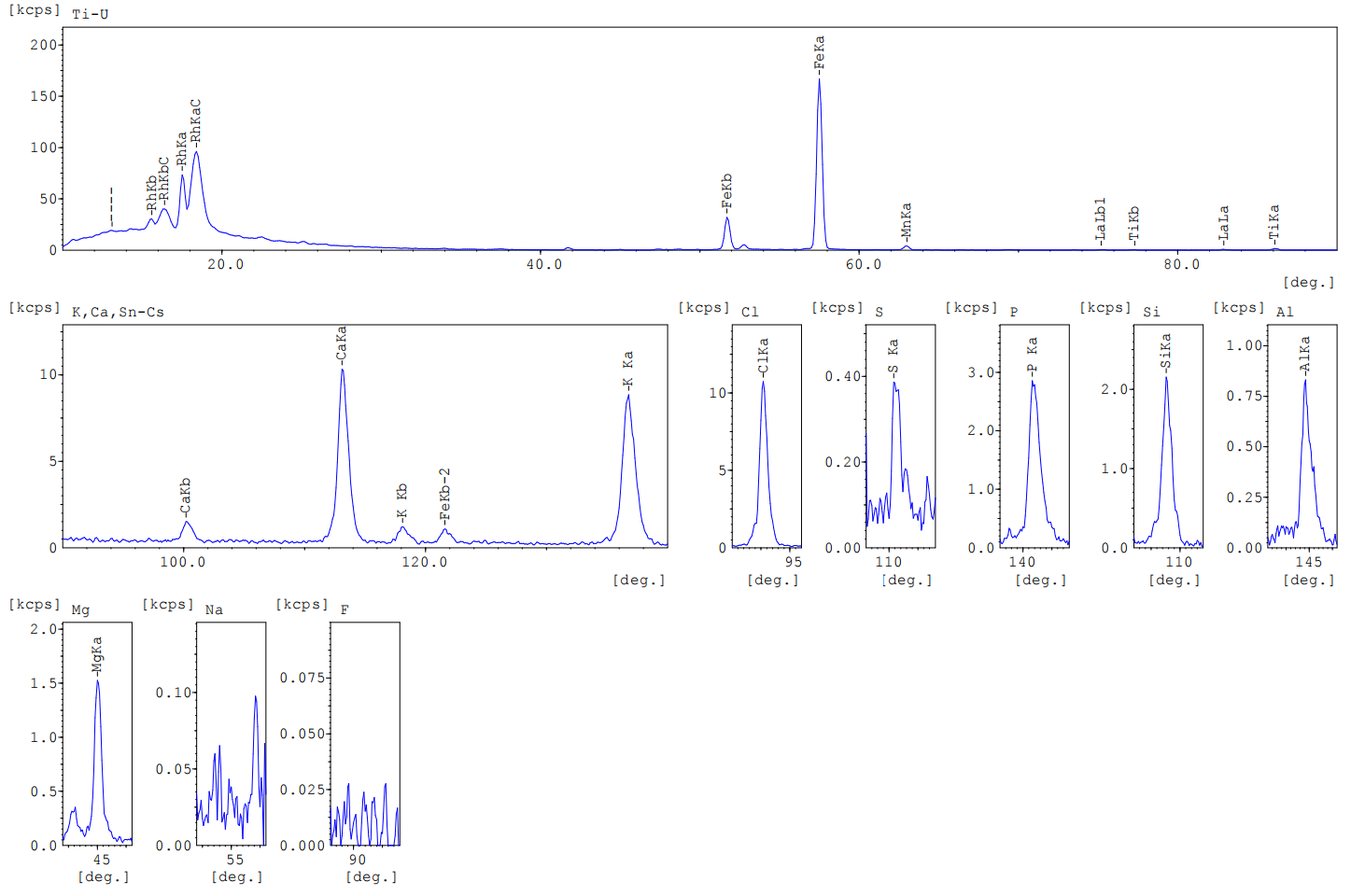

Supplement: S4 Fig — (TIF) [file pone.0311430.s004.tif]

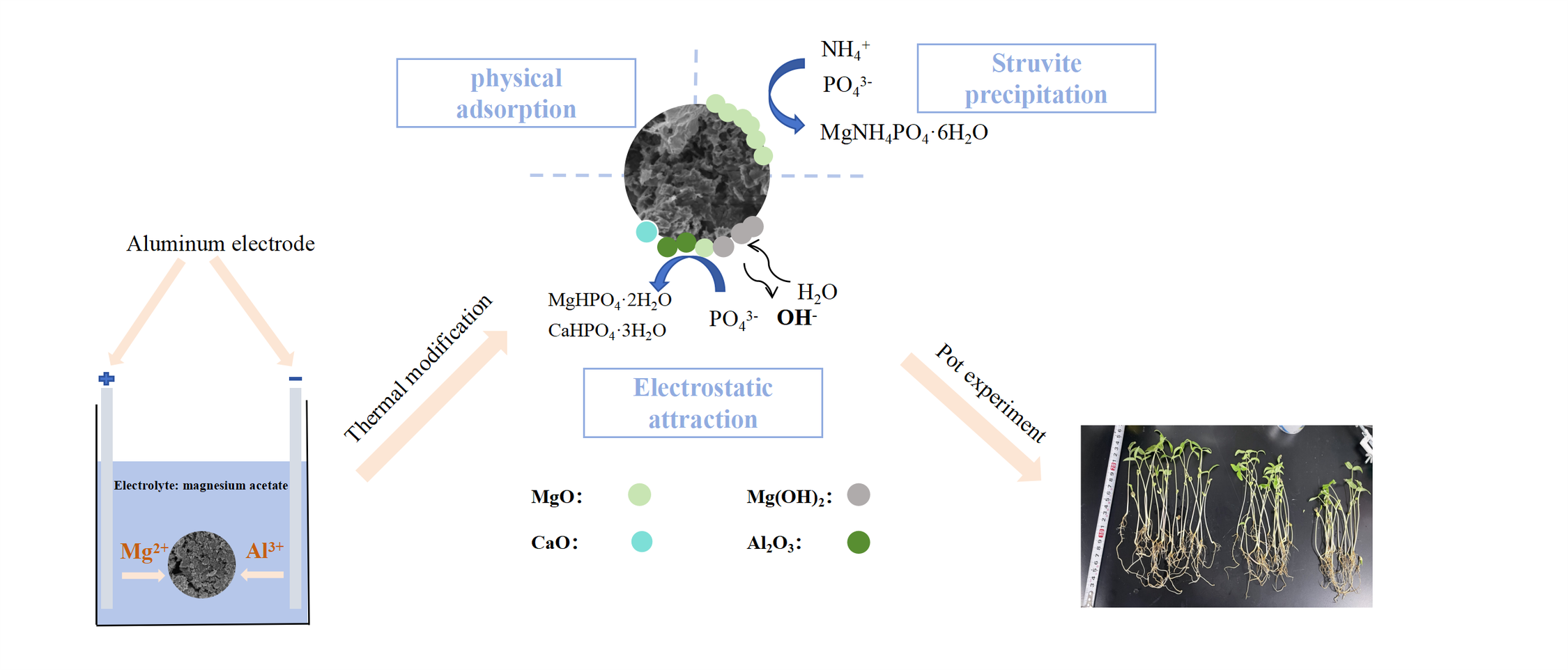

Supplement: S1 Graphical abstract — (TIF) [file pone.0311430.s005.tif]
