# Supplementary material for: Simultaneous adsorption of ammonia nitrogen and phosphate on electro-assisted magnesium/aluminum-loaded sludge-based biochar and its utilization as a plant fertilizer
Source: PLoS One. 2024 Oct 25;19(10):e0311430. doi: 10.1371/journal.pone.0311430 (PMC11508052; doi:10.1371/journal.pone.0311430)
Supplement: Attachment — Submitted filename: comments.docx [file pone.0311430.s006.docx]

This study prepared biochar for simultaneous removal of ammonium nitrogen and phosphate. To help the authors improve this manuscript, specific suggestions and comments are put forward as follows:

1. Please re arrange the introduction. For example; you should mentioned kind of materials used to make biochar, then explain what is biochar.

2. Is that enough to remove moisture content at 105 C for 2 hour?

3. Do you measure soil characteristics? Please add in the manuscript

4. Germination rate calculation? please add in the manuscript

5. Soil experiments under room temperature or constant temperature? Under greenhouse or field experiment? Please make a detail, because next researcher need to understand the methodology and developed it.

6. I think the title should be change by adding its application on plant
