# Supplementary material for: Simultaneous adsorption of ammonia nitrogen and phosphate on electro-assisted magnesium/aluminum-loaded sludge-based biochar and its utilization as a plant fertilizer
Source: PLoS One. 2024 Oct 25;19(10):e0311430. doi: 10.1371/journal.pone.0311430 (PMC11508052; doi:10.1371/journal.pone.0311430)
Supplement: Attachment — Submitted filename: Responses to Reviewers.docx [file pone.0311430.s007.docx]

**Response to Reviewer 1’s comments**

This study describes the production of a Mg/Al modified sludge-based biochar tested in the removal of phosphate and ammonia nitrogen from water. Despite some interesting results, several aspects should be improved and cleaned before a final decision. Specific comments are given below:
1. On page 13, line 3 of the ‘Batch adsorption experiments’ section, author mention that 'Mix a solution containing 50 mg·L^-1^ of PO_4_^3-^-P and 100 mg·L^-1^ of NH_4_^+^-N.' How did the authors determine the concentrations of the two pollutants and is there a correlation between the ratios of nitrogen and phosphorus? Please give the reasoning.
Thank you for your question. The reason why we mixed 100 mg·L^-1^ NH_4_^+^-N solution and 50 mg·L^-1^ PO_4_^3-^-P solution in the experiment is: At present, the research group undertook the project of “transformation and promotion of low-carbon sub-quality treatment technology of Rural domestic sewage in Water network/Hilly Area” in Jiangsu Province. In some rural areas of Jiangsu Province, the concentrations of NH_4_^+^-N and total phosphorus in the effluent of septic tanks were generally around 100 mg·L^-1^ and 40-60 mg·L^-1^. In order to better simulated the removal of NH_4_^+^-N and phosphorus in actual black water and promoted the sewage treatment of the project to achieve better results, the initial concentration of this experiment was selected in this way.

2. Why did author use H_2_SO_4_ for acidity adjustment in the 'Preparation of materials' section, but why use HCl for acidity adjustment in the 'Batch adsorption experiments' section?
   Thank you for your suggestion. We checked this area and found that it was a writing error, all of which were pH adjusted using HCl and NaOH.

3.In the section 'Batch adsorption experiments', 'Kinetic, isothermal and thermodynamic experiments', author do not give the experimental conditions such as pH value.
Thank you for your suggestion. We added relevant conditions and revised this paragraph to:

“Modified BC (0.02 g) was added to 30 mL of the adsorption solution, the pH was adjusted to 7, and the mixture was shaken at a speed of 150 rpm.”

“Adsorption experiments were conducted at 25°C, 35°C, and 45°C, and the pH of the solution was adjusted to 7 using HCl and NaOH.”

“Adsorption kinetics were also investigated. Specifically, 50 mg·L^−1^ PO_4_^3−^–P and 100 mg·L^−1^ NH_4_^+^–N solutions were mixed and stirred at 35°C and 150 rpm. The pH of the solution was adjusted to 7.”

4.In the section 'Effect of different factors on NH_4_^+^-N and PO_4_^3-^-P capture', page 30, line 16, there is an error in 'Fe_4_(PO_4_)_4_'.
Thank you for your suggestion. We changed this sentence to:

“Ca^2+^ and Fe^3+^ in the solution would immediately react with PO_4_^3−^–P to form amorphous CaCO_3_ and FePO_4_, respectively, producing irregular crystals and seriously hindering the crystallization of struvite.”

5.Page 36, 'Nitrogen and phosphorus recovery' section, lines 11-12, author suggest that 'Group A might promote the growth and development of mung bean sprouts because it absorbed rich N and P.' However, the ability of MgSBC-0.5(@Al)-NP to release N and P has not been studied in the manuscript, there is a lack of evidence to support this. It is suggested to supplement the related research on the performance of MgSBC-0.5(@Al)-NP in releasing N and P for a combined supplementary explanation.
Thank you for your suggestion. We added the leading experiment to study the NH_4_^+^-N and PO_4_^3-^-P release performance of MgSBC-0.5(@Al)-NP. The relevant contents are as follows:

Fig 8. (d) NH_4_^+^-N and PO_4_^3-^-P leaching rates of MgSBC-0.5(@Al)-NP and commercial fertilizers.

The leaching characteristics of MgSBC-0.5(@Al)–NP were different from those of commercial fertilizers. The release of NH_4_^+^–N and PO_4_^3−^–P in MgSBC-0.5(@Al)-NP showed a gradually increasing trend through the leaching process. When the L/S ratio reached 2.0 L·kg^-1^, the cumulative leaching amounts of NH_4_^+^–N and PO_4_^3−^–P were only 2.53 mg·g^-1^(6.98%) and 6.05 mg/g (8.21%). At the end of the experiment, the cumulative leaching amounts of NH_4_^+^–N and PO_4_^3−^–P were 11.54 mg·g^-1^(32.12%)and 26.36 mg·g^-1^ (35.78%), which were 2.72 times and 2.54 times lower than those of commercial fertilizers, respectively.

6. Do the Mg/Al ions in MgSBC-0.5(@Al) leach? How about the release? This can be discussed in conjunction with the corresponding emission standards.

Thank you for your advice. pH is the main factor affecting ion leaching, so we analyzed the leaching behavior of Mg/Al ions of MgSBC-0.5(@Al) elements in the pH range of 3~11. The intermittent leaching technique was used for experimental analysis according to the methods of the United States Environmental Protection Agency (USEPA 2014). The leaching concentrations of Mg^2+^ and Al^3+^ in eluent were analyzed by ICP-MS. When pH=3, the leaching amounts of Mg^2+^ and Al^2+^ were the highest, at 1.21 mg/L and 0.02 mg/L respectively. The state had no clear requirements for Mg^2+^ in groundwater, the evaluation was made according to the general content of tap water (magnesium content <300 ppm), which met the requirements of tap water. The amount of Al^3+^ leaching met the Chinese groundwater discharge Class II standard (< 0.05 mg/L).

**Response to Reviewer 2’s comments**

This study prepared biochar for simultaneous removal of ammonium nitrogen and phosphate. To help the authors improve this manuscript, specific suggestions and comments are put forward as follows:

1. Please re arrange the introduction. For example; you should mentioned kind of materials used to make biochar, then explain what is biochar.

Thank you for your suggestion. We re arranged the content of the introduction section. The overall writing idea is that: the current water pollution caused by PO_4_^3-^-P and NH_4_^+^-N. Definition and raw materials of biochar; Common modification methods and their drawbacks; The advantages and problems of electro-assisted modification method; The direction and content of our research. The second paragraph of the introduction section reads as follows:

“Biochar (BC) is a porous, loose, aromatic solid product formed through the pyrolysis of biomass under limited oxygen conditions. BC has a certain amount of surface functional groups, large specific surface area, wide availability of required raw materials, and low cost. Additionally, BC can adsorb pollutants from water [4]. Agricultural waste (such as straw, sawdust, sugarcane bagasse, and rice bran) and animal manure can be used as raw materials for the production of BC [5]. Some studies have shown that BC prepared from sludge exhibits strong adsorption capacity for organic and metal pollutants. Pyrolysis substantially reduces the ecological toxicity of heavy metals in sludge BC, reducing the environmental risks of its application [6]. Compared to BCs based on other types of biomass, sludge BC can be used to cost-effectively adsorb N and P. Therefore, sludge BC has unique advantages over other BC materials in practical applications [7]. However, the functionality and adsorption capacity of BC prepared via conventional pyrolysis are limited, making it unsuitable for pollutant adsorption. Consequently, BC is chemically modified, including impregnation modification with single metals (Fe, Mg, etc.) [8-10] and pairs of metals (Mg/Al, Ca/Mg, etc.) [11, 12]. Among the modified BCs, magnesium salt–modified biochar has high surface activity, anion fixation ability, and ion exchange ability. Magnesium-modified BC (Mg–BC) has been previously studied as a potential adsorbent for N and P. Mg–BC is prepared mainly through impregnation carbonization and carbonization impregnation. The modification of BC with Mg has been performed by soaking it in MgCl_2_ solution. However, the immersion time is long, usually more than 2 h [13]. Therefore, some researchers have proposed electro-assisted modification. Mg/Al bilayer metal–modified BC was prepared via an electro-assisted method using MgCl_2_ solution electrolyte and Al electrode, with an impregnation time of only 5 min. The formed compounds (MgO, spinel MgAl_2_O_4_, AlOOH, and Al_2_O_3_) evenly covered the surface of BC, exhibiting a highly organized and well-defined structure, resulting in improved PO_4_^3−^–P adsorption capability of BC.”

2. Is that enough to remove moisture content at 105 ℃ for 2 hour?

Thank you for your question. After verification, due to the low water content of the sludge we selected, it could be dried for about 2-3 hours at 105 ℃. In order to be accurate and convenient for subsequent researchers, we changed the original text to: “After anaerobic fermentation, the sludge was dried in an oven at 105°C to remove moisture content, ground to a fine powder (smaller than 200 mesh), and stored in a dry place.”

3. Do you measure soil characteristics? Please add in the manuscript

Thank you for your suggestion. We tested the basic properties of the original soil and supplemented it in the text

**Table 1. Basic properties of soil.**

|  | Moisture content | pH | CEC | organic matter | Total phosphorus | Total nitrogen | Available Phosphorus | Hydrolyzable nitrogen |
| --- | --- | --- | --- | --- | --- | --- | --- | --- |
| Primitive soil | % |  | (cmol·kg^-1^) | (g·kg^-1^) | (g·kg^-1^) | (g·kg^-1^) | (mg·kg^-1^) | (mg·kg^-1^) |
|  | 11.7 | 7.72 | 18.86 | 18.9 | 0.559 | 0.882 | 24.20 | 49 |

4. Germination rate calculation? please add in the manuscript

The germination rate (GP; %) of mung bean sprouts was calculated using Eq. (12).

| $\text{Germination percentage (GP)=}\frac{\text{n}}{\text{N}}\times\text{100\%}$ | （12） |
| --- | --- |

where *n* is the number of seeds germinated on the fourteenth day, and *N* is the total number of seeds.

5. Soil experiments under room temperature or constant temperature? Under greenhouse or field experiment? Please make a detail, because next researcher need to understand the methodology and developed it.

Thank you for your question. Our soil experiment was conducted under constant temperature in a greenhouse (with a temperature maintained at around 25 °C). The corresponding section in the article has been modified as follows：

“Five mung beans were planted in each pot and allowed to grow in a greenhouse at a temperature of around 25°C. Each grid was watered with 2.5 mL of tap water every day.”

6. I think the title should be change by adding its application on plant

Thank you for your suggestion. We revised the title as follows:

“Simultaneous adsorption of ammonia nitrogen and phosphate phosphorus on electro-assisted magnesium/aluminum-loaded sludge-based biochar and its utilization as a plant fertilizer”

**Response to Reviewer 3’s comments**

The manuscript entitled “Electro-assisted loading of magnesium/aluminium onto sludge-based biochar for the simultaneous adsorption of ammonia nitrogen and phosphate” by Qi Wang et al. presents the modification of sewage sludge with Mg and Al through electrochemical methods. The Mg/Al modified sewage sludge is then pyrolyzed and the modified biochar is used for simultaneous adsorption of ammonium and phosphate. The premise of the study is quite unique because electrochemical modification is not commonly used for biochar modification. The manuscript has several major drawbacks:

(i) The mechanism of the electrically assisted impregnation is not clearly explained. To what extent does the impregnation method affect the biochar properties, and what is the mechanism?

Thank you for your question. We found relevant literature and provided explanations, as well as supplemented the mechanism explanation section.

Through electrically assisted impregnation, the porosity of biochar could be effectively improved by oxidizing strong oxidants (providing more chemical binding sites) in about 10 minutes. In this experiment, Mg^2+^ and Al^3+^ could be loaded onto biochar to enhance its physical and chemical adsorption capabilities.

The adsorption capacities of NH_4_^+^–N and PO_4_^3−^–P on biochar (MgSBC-0.5(@Al) impregnated with electrically assistance at 298k were 65.19 mg·L^-1^ and 92.10 mg·L^-1^, respectively. The adsorption capacities for NH_4_^+^–N and PO_4_^3−^–P were 5.92 and 6.28 times than those of biochar without impregnation modification (SBC).

(ii) The authors have prepared only two biochars (one with Mg/Al and one without). There is no variation of the impregnation conditions (e.g., impregnation time, current density) and pyrolysis conditions (e.g., pyrolysis temperature). The authors did vary the Mg acetate concentration, but those biochars were only tested in the adsorption experiment and not characterized. Thus, the material preparation conditions and the adsorption performance cannot be clearly related.

Thank you for your suggestion. We supplemented the XRD characterization of biochar with different magnesium acetate concentrations and conducted correlation analysis.

Fig. S1 The XRD patterns of MgSBC-0.1(@Al), MgSBC-0.25 (@Al), MgSBC-0.5(@Al), and MgSBC-1(@Al)

“The four materials were characterized by XRD. It can be seen from S1b Fig that the peak of MgO in MgSBC-0.5(@Al) is higher than that of the other three materials, indicating that MgSBC-0.5(@Al) is loaded with more MgO.”

(iii) The role of aluminum is not clear. What was the role of Al in the adsorption of ammonium and phosphate? Or did the Al have a different role?

Thank you for your question. We reviewed the literature and found that the loading of Al^3+^could promote the enrichment of porous structures in biochar, which was beneficial for its application as an adsorbent. And some scholars have proven that metal ions (i.e. Al^3+^, Mg2+ and Ca^2+^) in the solution could also precipitate with PO_4_^3-^-P through strengthened chemical bonds, effectively improving the adsorption capacity of PO_4_^3-^-P. Therefore, the content and morphological properties of metal oxides (such as Al_2_O_3_) had a substantial impact on the adsorption capacity of PO_4_^3-^-P. We added the following explanation for the effect of Al^3+^in the text:

“The loading of Al_2_O_3_ was beneficial for the formation of porous structures in biochar. The presence of Al^3+^ in solution could precipitate with PO_4_^3-^-P through strengthened chemical bonds. This might improve the adsorption capacity of MgSBC-0.5(@Al) for PO_4_^3-^-P [20]”

(iv) The main adsorption mechanism of ammonium and phosphate is explained to be due to struvite crystallization and formation of MgHPO_4_ and CaHPO_4_, but insufficient evidence is given. Such claims should be supported by a more in-depth characterization of the post-adsorption material (e.g., XRD measurements) supported by solubility calculations.

We supplemented XRD characterization and found diffraction peaks of MgHPO_4_ at 2θ=27.81 ° and 18.51 °. However, due to the low content of CaHPO_4,_ it could not be accurately identified in XRD. Furthermore, diffraction peaks of MgNH_4_PO_4_·6H_2_O were detected at 2θ=0.85 ° (111), 21.45 ° (021), 25.72 ° (200), and 30.19 ° (012), indicating the presence of MgNH_4_PO_4_·6H_2_O and MgHPO_4_ in MgSBC-0.5(@Al)-NP

Fig 6. (b) XRD spectra of MgSBC-0.5(@Al) adsorption of NH_4_^+-^-N and PO_4_^3-^-P.

(v) A plant study was conducted to test the performance of the post-adsorption biochar as a soil amendment. This experiment has several problems namely (1) the number of replicates is not stated, (2) no positive control group was included, (3) no statistical analysis was carried out, and (4) the N and P contents in the sprouts were not measured.

(1)Each group of experiments was carried out four times, which had been added in the paper.

(2)Positive control group D (commercial fertilizer) was added. Fig 8. (a-b) Growth of mung beans on days 1 and 14, (c) Root length, stem length, average wet weight and average dry weight of five groups of mung beans on day 14, and (d) NH_4_^+^-N and PO_4_^3-^-P leaching rates of MgSBC-0.5(@Al)-NP and commercial fertilizers.

(3)We conducted a statistical analysis as follows.

-Thank you for your suggestions. We used SPSS statistics software to conduct substantially difference analysis on the four indicators, and the results are as follows,

After SPSS Statistics calculation, for stem length, average wet weight and average dry weight, there were substantially differences between groups A and E. And Group A was substantially different from the other three groups (Groups B, C, and D) (p<005). But for Root length, There was only a substantially difference between groups C and D. For mung bean sprouts, the application of commercial fertilizer (group D) had a great impact on the four indicators, and the effect was better than that of group A. But the application of group A (MgSBC-0.5(@Al)-NP) substantially affects the stem length, average wet weight, average dry weight, and average dry weight of mung bean sprouts than those of group B, C and E (Table 9).

(4)Thank you for your suggestion. We used Kjeldahl nitrogen analyzer and inductively coupled plasma emission spectrometer to detect the content of nitrogen and phosphorus in group A, B and C mung bean sprouts (14 days). According to the detection method of NY/T 2017-2011, nitrogen and phosphorus contents in mung bean sprouts of group A samples were 41.28 mg·kg^-1^ and 3.856 mg·kg^-1^, respectively. It could be concluded that the addition of MgSBC-0.5(@Al)-NP would slightly increase the nitrogen and phosphorus contents in mung bean sprouts.

Table.9 Effects of five groups on yield and characters of mung bean sprouts.

| Group | Stem Length (cm) | Root Length (cm) | Average wet weight  (g) | Average dry weight  (g) | Nitrogen  (g·kg^-1^) | Phosphorus  (g·kg^-1^) |
| --- | --- | --- | --- | --- | --- | --- |
| A | 12.58 ± 3.33b | 9.15 ± 4.10ab | 0.42 ± 0.041b | 0.085 ± 0.0068b | 41.28 | 3.86 |
| B | 11.52 ± 2.52c | 8.21 ± 3.17ab | 0.36 ± 0.029c | 0.069 ± 0.0089c | 30.07 | 2.21 |
| C | 8.92 ± 3.89c | 7.02 ± 2.54b | 0.32 ± 0.024d | 0.066 ± 0.0038cd | 29.12 | 2.16 |
| D | 16.79 ± 1.41c | 9.34 ± 2.32a | 0.65 ± 0.035a | 0.092 ± 0.0080a | 57.17 | 5.96 |
| E | 8.58 ± 0.92a | 7.99 ± 1.38 ab | 0.34 ± 0.033c | 0.063 ± 0.0048d | 29.66 | 2.17 |

Note：1:Repeat 4 times for each group of samples; 2: Values with superscript letters a, b, c, and d are significantly different across columns (p<005).

(vi) Presentation and discussion of the results is confusing, both due to the language used and conclusions drawn based on the presented (insufficient) data.
More specific comments to improve the manuscript are given below:

1. Introduction: “Magnesium (Mg)/aluminum (@Al) bilayer metal … adsorption capability of BC”. Please provide a reference to this study.

Thank you for your question. Referring to the research method of literature “Jung KW, Jeong TU, Hwang MJ, Kim K, Ahn KH. Phosphate adsorption ability of biochar/Mg-Al assembled nanocomposites prepared by aluminum-electrode based electro-assisted modification method with MgCl_2_ as electrolyte. Bioresource Technology. 2015;198:603-10. Epub 2015/10/04. doi: 10.1016/j.biortech.2015.09.068. PubMed PMID: 26433157.”. This article used seaweed as the biochar, MgCl_2_ as the electrolyte, and an effective electroactive modification method to prepare biochar containing Mg/Al nanocomposites. The structural properties and phosphate adsorption performance of the biochar were studied. The maximum adsorption capacity of the phosphate was as high as 887 mg/g. The author demonstrated that this novel electrically assisted modification was a highly attractive method for preparing Mg/Al biochar nanocomposites.

2. Introduction: “When PO_4_^3-^-P, Mg^2+^, and NH_4_^+^-N are combined in solution, NH_4_^+^-N and PO_4_^3-^-P can be removed.”. Why is this? Please clarify.

Thank you for your question. We referred to many relevant literature, such as:

1. Thant Zin MM, Kim DJ. Simultaneous recovery of phosphorus and nitrogen from sewage sludge ash and food wastewater as struvite by Mg-biochar. Journal of Hazardous Materials. 2021;403:123704-12. Epub 2020/12/04. doi: 10.1016/j.jhazmat.2020.123704. PubMed PMID: 33264890.
2. Wang CY, Zhou HD, Wang Q, Xu BX, Zhu G. Efficiency and mechanism of phosphate adsorption and desorption of a novel Mg-loaded biochar material. Environmental Science and Pollution Research. 2024;31(3):4425-38. Epub 2023/12/16. doi: 10.1007/s11356-023-31400-z. PubMed PMID: 38102434.

When PO_4_^3-^-P, Mg^2+^, and NH_4_^+^-N were in solution, chemical reactions such as Struvite crystallization (MgNH_4_PO_4_·6H_2_O) and MgHPO_4_ occurred, thereby removing NH_4_^+^-N and PO_4_^3-^-P.

3. Introduction: “Therefore, sludge has unique advantages over other BC materials in practical application [11].”. It is not clear how the authors come to this conclusion based on the information given in the discussed paragraph. Please clarify.

Thank you for your suggestion. We removed “Therefore, sludge has unique advantages over other BC materials in practical application [11]” and added it to the second paragraph. We also explained why sludge was chosen for the preparation of biochar materials. The content is as follows:

“Some studies have shown that BC prepared from sludge exhibits strong adsorption capacity for organic and metal pollutants. Pyrolysis substantially reduces the ecological toxicity of heavy metals in sludge BC, reducing the environmental risks of its application [6]. Compared to BCs based on other types of biomass, sludge BC can be used to cost-effectively adsorb N and P. Therefore, sludge BC has unique advantages over other BC materials in practical applications [7].”

4. Materials and methods, preparation of materials: please explain where and how the sludge was collected.

Thank you for your question. We provided an answer to it.

- Location of sludge collection: aerobic tank of sewage treatment plant.
- Sludge treatment method: anaerobic fermentation treatment.

And the content has been modified in the Materials and Methods section as follows:

“After anaerobic fermentation, the sludge was dried in an oven at 105°C to remove moisture content, ground to a fine powder (smaller than 200 mesh), and stored in a dry place.”

5. Materials and methods, preparation of materials: “Subsequently, the initial pH was modified to 3.0”. Why was this done? Please clarify.
Thank you for your question. We referred the operating methods in literature (Jung KW, Jeong TU, Hwang MJ, Kim K, Ahn KH. Phosphate adsorption ability of biochar/Mg-Al assembled nanocomposites prepared by aluminum-electrode based electro-assisted modification method with MgCl_2_ as electrolyte. Bioresource Technology. 2015;198:603-10. Epub 2015/10/04. doi: 10.1016/j.biortech.2015.09.068. PubMed PMID: 26433157.)

The operation was carried out in this way in the text: Subse sequenced, a certain amount of the dried marine macroalgae was dipped in 100 mL of the prepared MgCl2 solution (solid/liquid ratio was fixed at 1:10) and then the initial pH was adjusted to 3.0 using 0.5 M H_2_SO_4_ and NaOH solutions. So in our experiment, the pH was also adjusted to 3 during the electro modification process. The reason is that under acidic conditions, Mg(OH)_2_ and Al(OH)_3_ do not dissolve and can be better loaded on the sludge. The precipitation of Al^3+^occurs at a pH of around 3.2, so the pH was adjusted to 3.

6. Materials and methods, preparation of materials: the experimental setup of the electrochemical modification is not clear. What was the anode material, and what was the cathode? Was a reference electrode used? Please clarify.

Thank you for your question. We added relevant information in the paragraph as follows:

“The anode and cathode in the electro-assisted modification device were both Al rods, and a saturated calomel electrode was used as a reference electrode.”

7. Materials and methods, preparation of materials: how were the NH_4_^+^-N and PO_4_^3-^-P solutions prepared?

Thank you for your suggestion, but based on relevant literature, there were no detailed explanation for the preparation of NH_4_^+^-N and PO_4_^3-^-P solutions. In this section of Materials and methods, the preparation of NH_4_^+^-N and PO_4_^3-^-P solutions were carried out in accordance with the 《National Environmental Protection Standards of the People's Republic of China》 (HJ535-2009 and HJ 670-2013). Therefore, due to the consideration of article length, the specific steps are not included in the main text, and the preparation method has been slightly added as follows:

“Solutions containing NH_4_^+^–N (measured in N units) and PO_4_^3−^–P at concentrations of 1000 mg·L^−1^ were prepared by dissolving appropriate amounts of NH_4_Cl and KH_2_PO_4_ in deionized water and diluting them to 1000 mL in a volumetric flask. Subsequently, the prepared stock solution was diluted to the desired concentration.”

8. Materials and methods, preparation of materials: please provide the purity and supplier of all reagents used.

Thank you for your question. The relevant information about the reagents is as follows:

| Reagent | Purity | Supplier |
| --- | --- | --- |
| Mg(CH_3_COO)_2_·4H_2_O | analytical pure | Shanghai Aladdin Biochemical Technology Co.,Ltd. |
| HCl | analytical pure | Sinopharm Chemical Reagent Co.,Ltd |
| NaOH | analytical pure | Sinopharm Chemical Reagent Co.,Ltd |
| Na_2_CO_3_ | analytical pure | Shanghai Aladdin Biochemical Technology Co.,Ltd. |
| Na_2_SO_4_ | analytical pure | Shanghai McLin Bio-chemical Technology Co. , Ltd. |
| NaCl | analytical pure | Sinopharm Chemical Reagent Co.,Ltd |
| NaHCO_3_ | analytical pure | Shanghai McLin Bio-chemical Technology Co. , Ltd. |
| FeCl_3_ | analytical pure | Shanghai McLin Bio-chemical Technology Co. , Ltd. |
| CaCl_2_ | analytical pure | Shanghai McLin Bio-chemical Technology Co. , Ltd. |

9. Materials and methods, batch adsorption experiments: the authors have carried out the adsorption experiments using a solution containing 50 mg·L^-1^ of PO_4_^3-^-P and 100 mg·L-1 of NH_4_^+^-N. How/why were these concentrations selected? Please clarify.

Thank you for your question. The reason why we mixed 100 mg·L^-1^ NH_4_^+^-N solution and 50 mg·L^-1^ PO_4_^3-^-P solution in the experiment is: At present, the research group undertook the project of “transformation and promotion of low-carbon sub-quality treatment technology of Rural domestic sewage in Water network/Hilly Area” in Jiangsu Province. In some rural areas of Jiangsu Province, the concentrations of NH_4_^+^-N and total phosphorus in the effluent of septic tanks were generally around 100 mg·L^-1^ and 40-60 mg·L^-1^. In order to better simulated the removal of NH_4_^+^-N and phosphorus in actual black water and promoted the sewage treatment of the project to achieve better results, the initial concentration of this experiment was selected in this way.

10. Please refer to all equations in the text.

Thank you for your suggestion. We reviewed equations throughout the text and added equations that were not cited. All equations were mentioned in the the text.

11. Please include the units of all symbols used in the equations.

Thank you for your suggestion, we checked the full text equations and added the missing units. All units of the equations are summarized in the table below.

| Symbol | Units |
| --- | --- |
| *Q*_t_ | mg·g^-1^ |
| *C*_0_ | mg·L^-1^ |
| *C*_t_ | mg·L^-1^ |
| *V* | mL |
| *m* | mg |
| *Q*_e_ | mg·g^-1^ |
| *C*_e_ | mg·L^-1^ |
| *k*_1_ | min^-1^ |
| *k*_2_ | g·mg^-1^·min^-1^ |
| *k*_id_ | mg/(g·min^1/2^） |
| *t* | min |
| *C* | / |
| *Q*_max_ | mg·g^-1^ |
| *K*_f_ | mg·(g·(L·mg^-1^)1/n)^-1^ |
| *K*_L_ | L·mg^-1^ |
| Δ*G*_0_ | kJ·mol^-1^ |
| Δ*H*_0_ | kJ·mol^-1^ |
| Δ*S*_0_ | kJ·mol^-1^·K^-1^ |
| *K*_C_ | / |
| *R* | 8.314 J·mol^-1^·K^-1^ |
| *T* | K |
| *M*_W_ | mol |

12. Materials and methods, batch adsorption experiments: “At the temperature of 35 °C, for the pH experiment”. Why was a temperature of 35 °C used for the pH experiment, while 25 °C was used for the previous experimentss?

Thank you for your suggestion. We checked the entire article and found that there were some typos. The previous experiment was also carried out at 35 °C, but it was written as 25 °C in the article. We standardized the experimental temperature in the article to 35 °C because the adsorption capacities of NH_4_^+^-N and PO_4_^3-^-P at 35 °C were higher than those at 25 ° C.

13. Equation 11: what is ‘D’? Please clarify.

Thank you for your question. After checking, we found that “D” represents desorption rate. We were originally planning to conduct NH_4_^+^-N and PO_4_^3-^-P desorption experiments, but after comparing various literature and application requirements, we ultimately chose to conduct soil experiments instead of desorption experiments. Currently, we deleted this formula from the article.

14. Materials and methods, pot experiments: here the authors mention both MgSBC-0.5(@Al) and MgSBC-0.5(@Al)-NP. What is the difference between these materials?

MgSBC-0.5(@Al)-NP is MgSBC-0.5(@Al) saturated with adsorbed NH_4_^+^-N and PO_4_^3-^-P. For the convenience of scholars to read, we added a sentence to this paragraph:“MgSBC-0.5(@Al) saturated with NH_4_^+^–N and PO_4_^3−^–P (hereafter referred to as MgSBC-0.5(@Al)–NP) was added to the soil at 0.5% of soil dry weight.”

15. Materials and methods, pot experiments: what is the “leaching process” that the authors are referring to here?

This “leaching process” refers to a subsequent leaching experiment designed to investigate the slow-release effects of MgSBC-0.5(@Al)-NP on NH_4_^+^-N and PO_4_^3-^-P. Relevant content and images were added to the article as follows:

Fig 8. (d) NH_4_^+^-N and PO_4_^3-^-P leaching rates of MgSBC-0.5(@Al)-NP and commercial fertilizers.

The leaching characteristics of MgSBC-0.5(@Al)–NP were different from those of commercial fertilizers. The release of NH_4_^+^–N and PO_4_^3−^–P in MgSBC-0.5(@Al)-NP showed a gradually increasing trend through the leaching process. When the L/S ratio reached 2.0 L·kg^-1^, the cumulative leaching amounts of NH_4_^+^–N and PO_4_^3−^–P were only 2.53 mg·g^-1^(6.98%) and 6.05 mg/g (8.21%). At the end of the experiment, the cumulative leaching amounts of NH_4_^+^–N and PO_4_^3−^–P were 11.54 mg·g^-1^(32.12%)and 26.36 mg·g^-1^ (35.78%), which were 2.72 times and 2.54 times lower than those of commercial fertilizers, respectively.

16. Materials and methods, pot experiments: the number of replicates in this experiment was not clear. Was only one pot used for each condition? Please clarify.

Thank you for your question. There are a total of 20 cells, and all groups were carried out four times for pot experiments.

17. Materials and methods, pot experiments: in each pot experiment both a negative control and positive control should be used. How did the performance of the MgSBC-0.5(@Al)-NP compare to a commercial fertilizer? And what was the performance of the raw sludge (SP) in the mung bean growth experiment?

Thank you for your advice. In the pot experiment, we added group D (adding commercial fertilizer) and group E (adding SP), respectively. The following figure showed the results of the experiment.

Fig 8. (a-b) Growth of mung beans on days 1 and 14, (c) Root length, stem length, average wet weight and average dry weight of five groups of mung beans on day 14, and (d) NH_4_^+^-N and PO_4_^3-^-P leaching rates of MgSBC-0.5(@Al)-NP and commercial fertilizers.

18. Materials and methods, characterization and analytical method: how were the samples prepared for ICP-MS measurements? Please clarify.

Thank you for your question. The preparation steps for our ICP-MS measurement sample were as follows: 2 mL sulfuric acid and 7 mL phosphoric acid were microwave heated at 150 ℃ for 10 minutes. After that, wait for 5 minutes at 180 °C and 0.5 hour at 200 °C. Place the sample on an acid analyzer to reach about 1mL, then transfer it to a 25 mL colorimetric tube and dilute it to 25 mL with deionized water for testing.

The working conditions for the determination of elements by inductively coupled plasma emission spectroscopy were as follows: The power of the instrument is 1150 W. Plasma gas flow is 12.5 L/min. The auxiliary air flow is 0.5 L/min. The flow rate of atomizer is 0.5 L/min. Peristaltic pump speed 45 rpm.

19. Results and Discussion, characterization and morphology: “Fig 1d shows the energy spectrum of MgSBC-0.5(@Al),demonstrating that the nano flake mainly consisted of Mg, Al, Si, chlorine (Cl), and oxygen (O) [12]”.
- What “nano flake” are the authors referring to here?
- Which sample does Fig. 1d refer to?
- What was the carbon content in the EDX analysis? Given that the authors have prepared biochars, the material is expected to contain a substantial amount of carbon.

Thank you for your question. We answered the three questions you raised.

-The “nano flake” in this place has been checked for a typo, and we corrected this paragraph to:“Fig 1d shows the energy spectrum of MgSBC-0.5(@Al), demonstrating that the sample mainly consists of Mg, Al, Si, Cl, and O.”

-Fig.1d in this article refered to the sample of [MgSBC-0.5(@Al). We](mailto:MgSBC-0.5(@Al).We) changed the title of Fig.1 to:“Fig 1. (a) SEM images of SBC, SEM images of MgSBC-0.5(@Al) at different expansion ratios (b-c) and (d) EDS spectra of MgSBC-0.5(@Al) corresponding to SEM images.”

-The carbon content was estimated to be 14.34% through XPS characterization. But there was no separate detection in EDX.

20. Results and Discussion, characterization and morphology: “indicating that the electrical assisted modification could increase the S_BET_ of BC, thereby increasing the adsorption capacities of pollutants.” A higher S_BET_ may not necessarily lead to a higher adsorption capacity, because the S_BET_ is not the only factor that affects the adsorption process. In various situations, materials with a low S_BET_ have reached outstanding adsorption capacities because of the unique functional groups. The second part of the statement (“thereby…”) should be removed.

Thank you for your suggestion. The increase in S_BET_ would not directly lead to an higher adsorption capacity of biochar for pollutants. This statement was not appropriate in this section,. So we removed “thereby” from this section and changed this paragraph to:

“In particular, the *S*_BET_ of MgSBC-0.5 is substantially smaller than that of MgSBC-0.5(@Al), indicating that the electro-assisted modification increased the *S*_BET_ of BC.”

21. The number of significant figures should be checked and reduced in all tables. For example in Table 1: the measured pore volumes are not known with such precision.

Thank you for your question. We checked all the tables and their corresponding contents in the article, and standardized the numbers to 2 decimal places. As the correlation coefficient (R^2^) is relatively close, for the convenience of comparison, we kept it to 3 decimal places.

22. Results and Discussion, characterization and morphology: “The peak observed at a wavelength of 465 cm^-1^ in SBC attributed to the Si-O-Si vibration. The peak at 465cm^-1^ corresponded to the Si-O-Si or Al-O-Al vibration.” These two sentences can be combined into a single sentence.

Thank you very much for your suggestion. We merged these two sentences into one sentence and made synchronous modifications in this article.

“The peak observed at a wavelength of 465 cm^-1^ in SBC was attributed to the Si-O-Si or Al-O-Al vibration.”

23. Results and Discussion, characterization and morphology: “The results of FTIR further confirmed the existence of Mg and Al in MgSBC-0.5(@Al)”. This is not correct. The FTIR analysis cannot provide any information about the elemental composition. Please remove this statement.

Thank you very much for your feedback. We removed this statement.

24. Results and Discussion, adsorption kinetics and isotherm: at the beginning of this paragraph, the authors explain that they have prepared sludge-based biochar with varying Mg acetate solution concentrations. However, no explanation of these materials is given in the Materials and methods, and these materials have not been characterized. Please explain their preparation in the Materials and methods section, and provide a characterization of these biochar with the same techniques as MgSBC-0.5(@Al). This may provide insight into how the Mg acetate concentration affects the NH_4_^+^-N and PO_4_^3-^-P adsorption capacity.

Thank you for your suggestions. We added relevant explanations in the Materials and methods section as follows:

“MgSBC-0.1(@Al), MgSBC-0.25(@Al), and MgSBC-1(@Al) were prepared following the same procedure as MgSBC-0.5(@Al but using different magnesium acetate concentrations during electro-assisted modification-0.1, 0.25, and 1 mol/L, respectively.”

The preparation process of MgSBC-0.1(@Al), MgSBC-0.25(@Al), and MgSBC-1 (@Al) was the same as that of MgSBC-0.5(@Al), except that the concentration of magnesium acetate used in their electrochemical modification process is different from that of MgSBC-0.5(Al)(0.5 mol/L magnesium acetate), which were 0.1 mol/L, 0.25 mol/L, 1 mol/L, respectively.

We supplemented the XRD characterization of MgSBC-0.1(@Al), MgSBC-0.25 (@Al), MgSBC-0.5(@Al), and MgSBC-1(@Al), as shown in Fig. S. And an analysis was conducted as follows:

Fig. S1 The XRD patterns of MgSBC-0.1(@Al), MgSBC-0.25 (@Al), MgSBC-0.5(@Al), and MgSBC-1(@Al)

25. Results and Discussion, adsorption kinetics and isotherm: “However, a high concentration of Mg^2+^ could cause the transformation of struvite crystals into Mg_3_(PO_4_)_2_.” Please provide experimental evidence for this statement or otherwise remove it.

Thank you for your valuable suggestion. After reviewing the literature, we found that the generation of Mg_3_(PO_4_)_2_ requires specific Mg^2+^, PO_4_^3-^-P concentrations, and other conditions. Further characterization was needed to prove its existence, which was not closely related to the research content of this article. Therefore, we removed this sentence from the article

26. Table 5: please mention the adsorbent in the caption.
Thank you for your suggestion. Based on your suggestion, we changed the caption of Table 5 to the following:

“Adsorption kinetics parameters of MgSBC-0.5(@Al) adsorption of NH_4_^+^-N and PO_4_^3-^-P.”

27. Results and Discussion, Effect of different factors on NH_4_^+^-N and PO_4_^3-^-P capture: “The pHpzc values of MgO, AlOOH and Al_2_O_3_ were 12.0, 8.2 and 9.1, respectively [39, 40].” It is not clear why this is relevant here. Instead, what was the pHpzc of the adsorbents used in this study?

Thank you for your question. After testing, we found that the reason for writing “The pHpzc values of MgO, AlOOH and Al_2_O_3_ were 12.0, 8.2 and 9.1, respectively” before was because MgO,AlOOH and Al_2_O_3_ were loaded on biochar. It was believed that its pH_ZPC_ would affect the adsorption of NH_4_^+^-N and PO_4_^3-^-P at different pH. At present, it could be seen from the literature that the pH_zpc_ of MgSBC-0.5(@Al) could be generally measured. So we deleted this sentence. After testing, the pH_zpc_ of MgSBC-0.5(@Al) was 10.45. We add the following statement in the text:

“The zero point potential (pH_ZPC_) of MgSBC-0.5(@Al) was 10.45, indicating the presence of hydroxyl ligands on its surface, which facilitated the adsorption of PO_4_^3-^-P. When the initial pH>pHzpc, the surface of MgSBC-0.5(@Al) carried a negative charge. There was a repulsive force between PO_4_^3-^-P and MgSBC-0.5(@Al), which inhibited electrostatic adsorption. However, in this case, chemical interactions such as precipitation reactions between Mg^2+^and HPO_4_^2−^ might contribute to the removal of PO_4_^3-^-P.”

28. Results and Discussion, Effect of different factors on NH_4_^+^-N and PO_4_^3-^-P capture: “Simultaneously, the loss of NH_4_^+^ in the form of NH_3_ gas occurred at elevated pH levels. This led to a little decrease in the adsorption of NH_4_^+^-N”.
- What does “elevated pH levels” mean? Please specify the values.

We will change this to “when the pH rises to 11”

- How does loss of N via NH_3_ gas decrease the adsorption capacity? If NH_3_ gas would escape, wouldn’t this lead to an overestimation of the adsorption capacity?
Thank you for your question. We verified it. When the pH rose to about 11, a high pH corresponds to an increase in the concentration of hydroxide ions (OH^−^) in the solution. NH_4_^+^-N was mainly in the form of NH_3_·H_2_O. Therefore, the adsorption capacity of MgSBC-0.5(@Al) for NH_4_^+^-N was decreased. So the overall adsorption effect was decreased. Therefore, we modified the corresponding cause part of the text.

“When the pH rose to about 11, a high pH corresponded to an increase in the concentration of hydroxide ions (OH^−^) in the solution. NH_4_^+^-N was mainly in the form of NH_3_·H_2_O. Therefore, the adsorption capacity of MgSBC-0.5(@Al) for NH_4_^+^-N was weakened. So the overall adsorption effect was decreased.”

28. Results and Discussion, Effect of different factors on NH_4_^+^-N and PO_4_^3-^-P capture: “The existence of K^+^ and Na^+^ in the solution would compete with NH_4_^+^-N to form struvite potassium (KMgPO_4_·7H_2_O) and sodium magnesium PO_4_^3-^-P heptahydrate [NaMgPO_4_·7H_2_O].” In theory this is possible, but the solubility of K-struvite and Na-struvite may not be the same as that of regular struvite (depending on the pH). Please check the solubility of K-struvite, Na-struvite, and regular struvite at the pH of the experimental conditions used.

At pH around 7, the Ksp of KMgPO_4_·7H_2_O and NaMgPO_4_·7H_2_O are as follows.

KMgPO_4_·7H_2_O:Ksp =10^-12.2^ ;

NaMgPO_4_·7H_2_O: Ksp=10^-11.6^

30. Fig. 5a: What was the pH of the solutions after the adsorption experiments?

Thank you for your suggestion. We added the pH value of the solution after the experiment.

Fig 5. (a) Effect of initial pH of solution on adsorption of ammonia nitrogen and phosphorus by MgSBC-0.5(@Al).

31. Fig. 6: did the XPS signal of Al change after the adsorption experiment?

Thank you for your question. After verification, the XPS intensity of Al before and after adsorption changed from 31366.7 a.u to 15234.8 a.u. Due to the content of Al was relatively low, so it was not obvious in Fig 6. But the weakening of Al 2p signal at about 72.08 eV could also be seen, as shown in the figure below (inside red box):

Fig 6. (a-c) FTIR, XRD and XPS spectra of MgSBC-0.5(@Al) adsorption of NH_4_^+-^-N and PO_4_^3-^-P.

Fig. Screenshot of raw data of Al 2p signal peak before and after adsorption.

32. Results and Discussion, Nitrogen and phosphorus recovery:
    - Were the treatments significantly different? Please include a statistical analysis.
    - In Fig. 7 please include error bars for the wet weight
    - Please include dry weight data.

-Thank you for your suggestions. We used SPSS statistics software to conduct substantially difference analysis on the four indicators, and the results are as follows,

After SPSS Statistics calculation, for stem length, average wet weight and average dry weight, there were significantly differences between groups A and E. And Group A was significantly different from the other three groups (Groups B, C, and D) (p<005). But for Root length, There was only a significantly difference between groups C and D. For mung bean sprouts, the application of commercial fertilizer (group D) had a great impact on the four indicators, and the effect was better than that of group A. But the application of group A (MgSBC-0.5(@Al)-NP) significantly affects the stem length, average wet weight, average dry weight, and average dry weight of mung bean sprouts than those of group B, C and E.

Table.9 Effects of five groups on yield and characters of mung bean sprouts.

| Group | Stem Length (cm) | Root Length (cm) | Average wet weight  (g) | Average dry weight  (g) | Nitrogen  (g·kg^-1^) | Phosphorus  (g·kg^-1^) |
| --- | --- | --- | --- | --- | --- | --- |
| A | 12.58 ± 3.33b | 9.15 ± 4.10ab | 0.42 ± 0.041b | 0.085 ± 0.0068b | 41.28 | 3.86 |
| B | 11.52 ± 2.52c | 8.21 ± 3.17ab | 0.36 ± 0.029c | 0.069 ± 0.0089c | 30.07 | 2.21 |
| C | 8.92 ± 3.89c | 7.02 ± 2.54b | 0.32 ± 0.024d | 0.066 ± 0.0038cd | 29.12 | 2.16 |
| D | 16.79 ± 1.41c | 9.34 ± 2.32a | 0.65 ± 0.035a | 0.092 ± 0.0080a | 57.17 | 5.96 |
| E | 8.58 ± 0.92a | 7.99 ± 1.38 ab | 0.34 ± 0.033c | 0.063 ± 0.0048d | 29.66 | 2.17 |

Note：1:Repeat 4 times for each group of samples; 2: Values with superscript letters a, b, c, and d are significantly different across columns (p<005).

-Thanks for your suggestion, we added the dry weight data and the the error bar of mung bean sprouts.

33. Results and Discussion, Nitrogen and phosphorus recovery: “Therefore, MgSBC-0.5(@Al)-NP had the potential to replace N and P fertilizers.” This is not true, because no comparison is made with traditional N and P fertilizers. This statement should be removed.

Thank you for your suggestion. We deleted this sentence

34. Results and Discussion, Nitrogen and phosphorus recovery: what were the N and P contents in the seedlings?

Thank you for your suggestions. We consulted relevant literature and found that most of them were for the detection of nitrogen and phosphorus in plants, so we conducted an experiment on the effect of MgSBC-0.5(@Al)-NP on the content of nitrogen and phosphorus in plants. We used Kjeldahl nitrogen analyzer and inductively coupled plasma emission spectrometer to detect the content of nitrogen and phosphorus in group A, B, C, D and E mung bean sprouts (14 days). According to the detection method of NY/T 2017-2011.

Table.9 Effects of five groups on yield and characters of mung bean sprouts.

| Group | Stem Length (cm) | Root Length (cm) | Average wet weight  (g) | Average dry weight  (g) | Nitrogen  (g·kg^-1^) | Phosphorus  (g·kg^-1^) |
| --- | --- | --- | --- | --- | --- | --- |
| A | 12.58 ± 3.33b | 9.15 ± 4.10ab | 0.42 ± 0.041b | 0.085 ± 0.0068b | 41.28 | 3.86 |
| B | 11.52 ± 2.52c | 8.21 ± 3.17ab | 0.36 ± 0.029c | 0.069 ± 0.0089c | 30.07 | 2.21 |
| C | 8.92 ± 3.89c | 7.02 ± 2.54b | 0.32 ± 0.024d | 0.066 ± 0.0038cd | 29.12 | 2.16 |
| D | 16.79 ± 1.41c | 9.34 ± 2.32a | 0.65 ± 0.035a | 0.092 ± 0.0080a | 57.17 | 5.96 |
| E | 8.58 ± 0.92a | 7.99 ± 1.38 ab | 0.34 ± 0.033c | 0.063 ± 0.0048d | 29.66 | 2.17 |

Note：1:Repeat 4 times for each group of samples; 2: Values with superscript letters a, b, c, and d are significantly different across columns (p<005).

Add the following in the main text:

“The contents of nitrogen and phosphorus in mung bean sprouts in group A (41.28mg·kg^-1^ and 3.86 mg·kg^-1)^ were also higher than those in groups B, C and D.”

35. The authors mention several times that adsorption of ammonium and phosphate was due to struvite formation. However, no evidence is given on the formation of struvite. X-ray diffraction analysis of the biochars after adsorption should be carried out to confirm whether any struvite was formed.

Thank you for your suggestions. We characterized the adsorbed biochar by XRD and supplemented it in the original text.

“The XRD images of MgSBC-0.5(@Al) before and after NH_4_^+^-N and PO_4_^3-^-P adsorption are compared to further elucidated the adsorption mechanism (Fig 6b). It could seen that the MgO peak in the XRD spectrum of biochar is weakened. A series of XRD peaks appeared at 20.85 ° (111), 21.45 ° (021), 25.72 ° (200) and 30.19 ° (012) for MgSBC-0.5(@Al), corresponding to the MgNH_4_PO_4`_H_2_O diffraction peak. This indicatedthat the synthesis of struvite crystallization.”

Fig 6**.** (b) XRD spectra of MgSBC-0.5(@Al) adsorption of NH_4_^+-^-N and PO_4_^3-^-P.
